# Simple synthesis of multi-halogenated alkenes from 2-bromo-2-chloro-1,1,1-trifluoroethane (halothane)

**DOI:** 10.3762/bjoc.18.167

**Published:** 2022-11-21

**Authors:** Yukiko Karuo, Atsushi Tarui, Kazuyuki Sato, Kentaro Kawai, Masaaki Omote

**Affiliations:** 1 Faculty of Pharmaceutical Sciences, Setsunan University, 45-1 Nagaotoge-cho, Hirakata, Osaka 573-0101, Japan

**Keywords:** aryl 1-monofluorovinyl ether, electrophilic 1,1-difluoroethene, halothane, multi-halogenated alkene, phenol

## Abstract

A series of aryl fluoroalkenyl ethers that contain chlorine and bromine as well as fluorine atoms were prepared in moderate to good yields via the reactions of phenols and 2-bromo-2-chloro-1,1,1-trifluoroethane (halothane) in the presence of KOH. This simple reaction enabled the construction of highly halogenated compounds with the potential for further functionalization. The reaction involved a highly reactive difluoroethylene intermediate, which was produced by the reaction between halothane and KOH.

## Introduction

**Figure 1:**
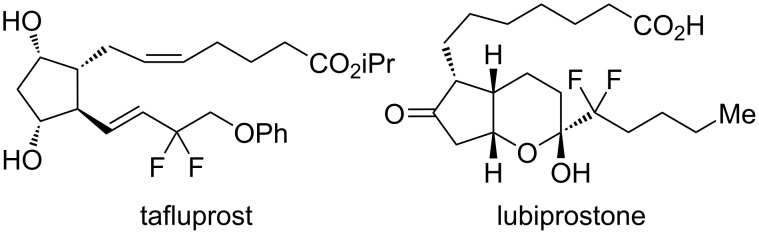
Medicines containing a difluoromethylene group.

**Scheme 1:**
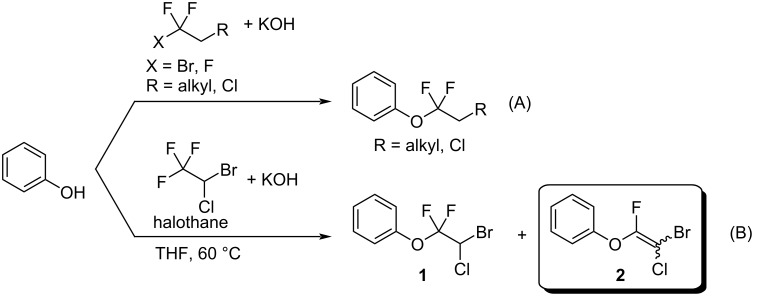
Reaction of phenol with polyfluoroalkanes.

2-Bromo-2-chloro-1,1,1-trifluoroethane (halothane) has been used as a fluorine-containing building block for the construction of trifluoromethyl and difluoromethylene motifs [1–2]. Such structures have been found in several multifunctional materials and biologically important molecules (Figure 1) [3–7]. The halothane structure contains two highly halogenated carbon centers, which enable halothane to participate in various reactions such as homolysis of carbon–halogen bonds and deprotonation. Multi-fluorinated compounds such as HCFC-133a (CF_3_CH_2_Cl) and HFC-134a (CF_3_CH_2_F) have been widely used in reactions with a variety of nucleophiles to afford 1,1-difluoro-2-haloethyl ethers, although their boiling points are below 6 °C, which often causes handling problems (Scheme 1A) [8]. 1,1-Difluoro-2-haloethyl ethers have been obtained by reacting HCFC-133a with alcohols in the presence of a small amount of water, but the reaction requires the use of a steel autoclave at 250 °C [9]. Other halogenated compounds that are not classified as freons have also been used for this type of fluoroalkylation [10–13].

**Figure 2:**
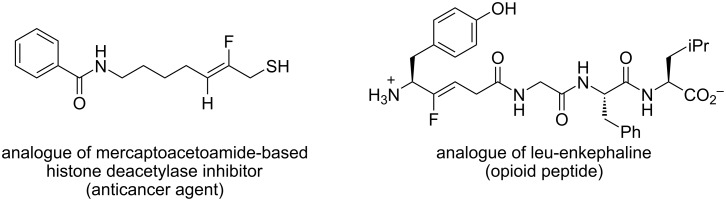
Fluoroalkene analogs of some drugs.

We recently reported that the reaction of halothane with various phenoxides proceeded smoothly to provide various aryl difluoroalkyl ethers (**1**), along with small amounts of fluoroalkenyl ethers (**2**), which were obtained from **1** via an E2-elimination mechanism (Scheme 1B) [14–15]. The fluoroalkenyl group in **2** is a potentially useful moiety that could participate in cross-coupling reactions for replacement of the bromine atom with an aryl or alkynyl unit [16–22]. In a biological context, the fluoroalkenyl motif could be incorporated into peptides with the expectation that the fluoroalkenyl unit could serve as a peptide isostere and prevent unexpected hydrolysis of these compounds. Recent studies have shown that the biological activities and metabolism properties of some fluoroalkenes are more potent than those of their parent compounds (Figure 2) [23–25]. To the best of our knowledge, only one report of highly halogenated aryl fluoroalkenyl ethers similar to **2** has been published [8]. In this report, **2** was treated as a byproduct and was not discussed in detail. The synthesis of such compounds remains a challenging task. We therefore tackled this issue of synthesizing highly halogenated alkenyl ethers. Here, we explored the synthesis of highly halogenated aryl fluoroalkenyl ethers **2** by using halothane as a halogen and carbon source.

## Results and Discussion

First, we optimized the reaction conditions for the formation of aryl fluoroalkenyl ethers with phenol (**3a**) as a model substrate. On the basis of our previous work, we performed the reaction of halothane with **3a** under the standard conditions for obtaining **1**. The desired highly halogenated aryl alkenyl ether **2a** was obtained, but the yield was unacceptably low (Table 1, entry 1). The low conversion is attributed to use of an insufficient amount of KOH, which was used as a base for deprotonation of the phenolic hydroxy group and acidic C–H bond between the bromine and chlorine atoms in **1**. Extra KOH was added to improve deprotonation, but the yield of **2a** was still low (Table 1, entries 2 and 3). Changing the solvent from THF to DME and increasing the temperature to 80 °C slightly improved the yield of **2a** to 19% (Table 1, entry 4). Decreasing the amount of halothane clearly increased the reaction efficiency to give **2a** in 69% yield (Table 1, entry 5). We reasoned that the reaction was largely dependent on the concentration of phenoxide ions, as these would act as both a nucleophile and base in the reaction medium to give formation of **1** and **2**. Halothane is so acidic that the basic KOH and phenoxide ions would be neutralized by the acidic hydrogens of halothane; this hindered the reaction in the cases of entries 1–4 in Table 1. The best result (Table 1, entry 6) was achieved by pretreating **3a** with KOH at room temperature for 1 h to convert **3a** completely to phenoxide ions. Halothane was then added to the reaction mixture, and the solution was heated to 80 °C for 4.5 hours. This optimized the reaction efficiency and **2a** was obtained in 85% yield. With the optimum conditions in hand, we investigated the scope and limitations of this reaction to explore the generality of this method.

**Table 1:** Optimization of reaction conditions for obtaining **2a** from **3a** and halothane.

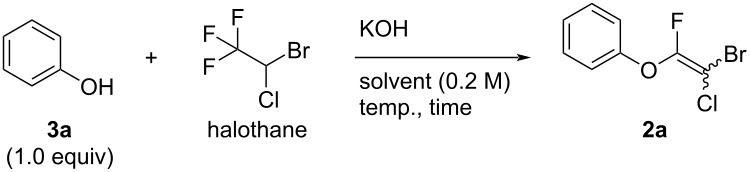						

Entry	Halothane (equiv)	KOH (equiv)	Solvent	Temp. (°C)	Time (h)	Yield of **2a** (%)^a^

1	2.0	1.5	THF	60	4.5	5
2	2.0	3.0	THF	60	4.5	3
3	2.0	5.0	THF	60	4.5	8
4	2.0	5.0	DME	80	4.5	19
5	1.0	5.0	DME	80	4.5	69
6	1.0	5.0	DME	rt to 80	5.5	85

^a^Isolated yield.

Initially, we performed the reaction with a small excess of 1-naphthol (**3b**, 1.05 equiv) relative to halothane (1.0 equiv), and used the same procedure as for Table 1, entry 6. The reaction proceeded smoothly to give 1-fluoro-2-bromo-2-chloroethenyl ether **2b** in 70% yield (Table 2, entry 1). The reaction under these conditions tolerated phenyl-substituted phenols and afforded **2c–e** in moderate to good yields (Table 2, entries 2–4). Electron-donating substituents, e.g., those in **3f** (4-OMe) and **3g** (2-*t-*Bu), did not compromise the reaction performance. However, the substituent bulkiness affected the reaction to some extent and the yield of **2g** was relatively low (Table 2, entries 5 and 6). In contrast, electron-withdrawing substituents were less compatible with the reaction, and **2h** (3-CF_3_) and **2i** (4-NO_2_) were obtained at significantly lower yields. This incompatibility with electron-withdrawing substituents can be attributed to lower nucleophilicity of the phenoxide ion and structural instability of the product, which could lead to unexpected reactions of **2h** and **2i** during the reaction or purification process. Phenols with allyl or vinyl substituents (**3j** and **3k**, respectively), which are susceptible to basic conditions, were tolerated in the reaction, but *ortho* substituents hindered the reaction to some extent (Table 2, entries 9 and 10). 2-Hydroxychalcone (**3l**), which has an electrophilic enone structure, was also tolerated. The Michael addition product was not detected (Table 2, entry 11). An aldehyde group was found to be incompatible with this method (Table 2, entry 12). Esters, which are susceptible to hydrolysis, can be used in the reaction under controlled conditions, i.e., with the temperature kept below 60 °C and THF as the solvent. However, the yield of **2n** was 32% (Table 2, entry 13). In the case of aminophenol (**3o**), nucleophilic addition occurred predominantly at the phenoxide position and the product was obtained in moderate yield (Table 2, entry 14). An aryl iodide also participated in the reaction (Table 2, entry 15).

**Table 2:** Scope of reaction with various substituted phenols (**3b–p**).

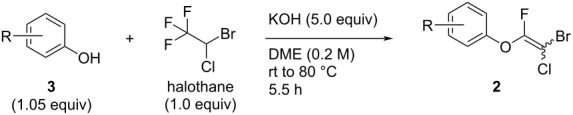			

Entry	Substrates	Products	Yield of **2** (%)^a^

1	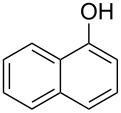 **3b**	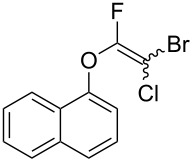 **2b**	70
2	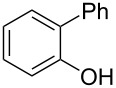 **3c**	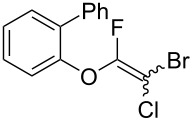 **2c**	85
3^b^	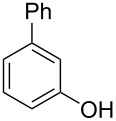 **3d**	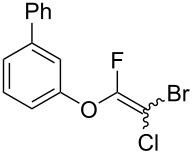 **2d**	68
4^b^	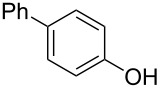 **3e**	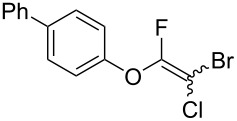 **2e**	63
5	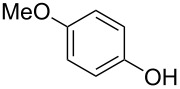 **3f**	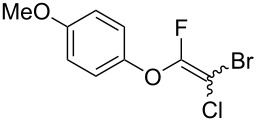 **2f**	76
6	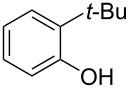 **3g**	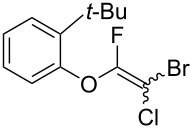 **2g**	41
7^c^	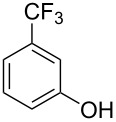 **3h**	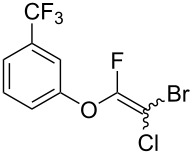 **2h**	39
8^c^	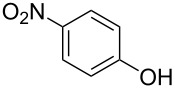 **3i**	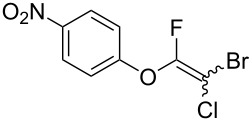 **2i**	18
9	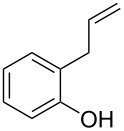 **3j**	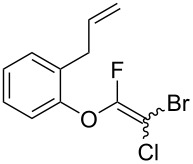 **2j**	75
10^c^	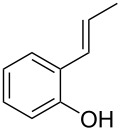 **3k**	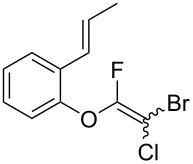 **2k**	54
11	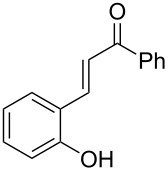 **3l**	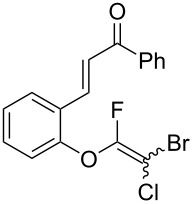 **2l**	51
12^d^	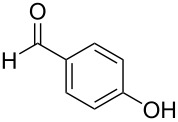 **3m**	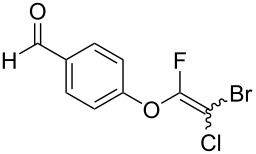 **2m**	9
13^e^	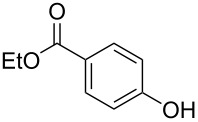 **3n**	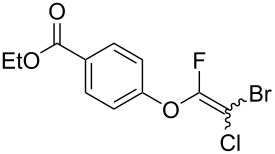 **2n**	32
14^f^	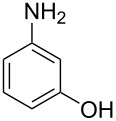 **3o**	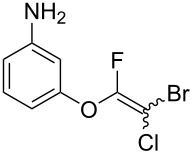 **2o**	66
15^b,g^	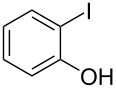 **3p**	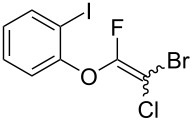 **2p**	69

^a^Isolated yield; ^b^**3** (1.0 equiv) was used; ^c^DME (0.5 M) was used; ^d^reaction time was 6.5 h; ^e^THF was used instead of DME; ^f^**3** (1.2 equiv) was used; ^g^halothane (1.5 equiv) was used.

**Scheme 2:**
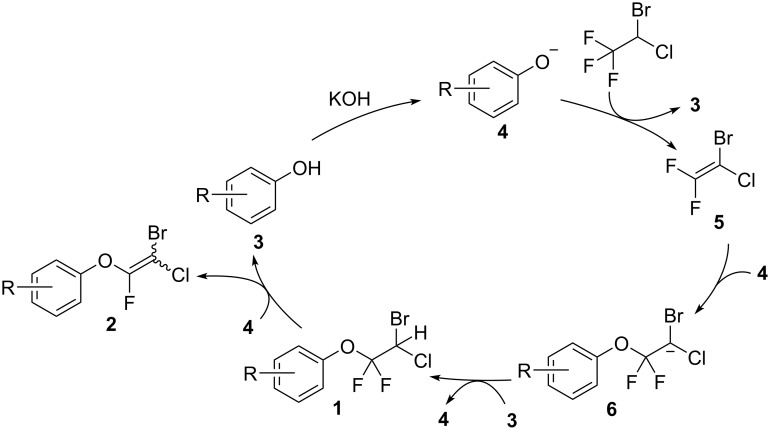
Proposed mechanism.

We propose the reaction mechanism shown in Scheme 2 [15,26]. In the reaction medium, **3** is deprotonated by KOH to generate phenoxide ion **4**, which acts as a base and as a nucleophile. Removal of an acidic hydrogen from halothane provides **5**, which is a key intermediate in the reaction. Intermediate **5** is sufficiently electrophilic to react with **4** because the carbanion **6**, which is generated from **4** and **5**, is less basic because of the double induction effect of the two halogen atoms. In this cycle, **1** can be neutralized in the reaction medium by proton sources such as **3**, halothane, and H_2_O. Dehalogenation of intermediate **6** or removal of HF from **1** provides **2** as an *E*/*Z* mixuture (*E*/*Z* = 1:1). We speculated that the stability of the *E* isomer was equal to that of the *Z* isomer under these conditions.

**Scheme 3:**
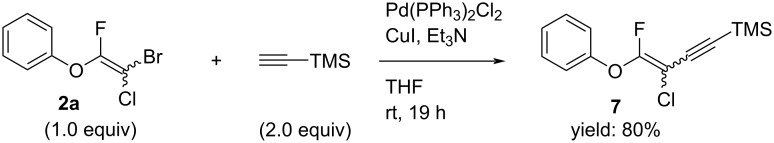
Sonogashira cross-coupling reaction of **2a** with trimethylsilylacetylene.

To expand the scope of this reaction, we subjected product **2** to a Sonogashira cross-coupling reaction (Scheme 3). This gave a highly functionalized enyne structure that will be useful in various molecular transformations [27–29]. On the basis of a previous report, Sonogashira cross-coupling of **2** with trimethylsilylacetylene was performed with a bis(triphenylphosphine)palladium(II) dichloride. The reaction proceeded smoothly to give highly functionalized **7** in 80% yield. Compound **7** contains an enyne motif with both haloalkene and alkenyl ether moieties.

## Conclusion

We have developed a simple method for constructing 1-fluoro-2-bromo-2-chloroalkenyl ethers (**2a**–**p**) in moderate to good yields via reactions of phenols and halothane in the presence of KOH. In this reaction, halothane plays a key role in the construction of highly halogenated and structurally intriguing products. The tri-halogenated alkenyl ether has potential applications in organic chemistry, e.g., in Suzuki–Miyaura or Sonogashira cross-coupling reactions. Further experiments with the aim of identifying further applications of **2** will be reported in due course.

## Experimental

### General information

^1^H NMR, ^19^F NMR and ^13^C NMR spectra were recorded on JEOL ECZ 400S spectrometers. Chemical shifts of ^1^H NMR are reported in ppm from tetramethylsilane (TMS) as an internal standard. Chemical shifts of ^13^C NMR are reported in ppm from the center line of a triplet at 77.16 ppm for deuteriochloroform. Chemical shifts of ^19^F NMR are reported in ppm from CFCl_3_ as an internal standard. All data are reported as follows: chemical shifts, relative integration value, multiplicity (s = singlet, d = doublet, t = triplet, q = quartet, sep = septet, br = broad, brd = broad-doublet, m = multiplet), coupling constants (Hz). Mass spectra were obtained on JEOL JMS-700T spectrometer (EI).

### Materials

All commercially available materials were used as received without further purification. All experiments were carried out under argon atmosphere in flame-dried glassware using standard inert techniques for introducing reagents and solvents unless otherwise noted.

### Typical procedures for synthesis of multi-halogenated alkene

Ground KOH (5.0 mmol) was added to a solution of phenol (1.0 mmol) in DME (5.0 mL). The mixture was stirred for 1 h at room temperature, and then halothane (1.0 mmol) was added in small portions. The solution was heated to 80 °C, and the temperature was maintained for 4.5 h. The reaction mixture was quenched by addition of saturated aqueous NH_4_Cl (40 mL) at 0 °C and extracted with Et_2_O. The organic phase was dried over Na_2_SO_4_, filtered, and concentrated by evaporation under reduced pressure. The residue was purified by column chromatography to afford **2**.

**2-Bromo-2-chloro-1-fluoroethenyl phenyl ether (2a):** Product **2a** was purified by column chromatography (pentane only). **2a** was obtained in 85% yield (212.7 mg) as a colorless oil. ^1^H NMR (400 MHz, CDCl_3_) δ 7.06–7.13 (2H, m), 7.16–7.23 (1H, m), 7.38 (2H, t, *J* = 7.7 Hz); ^13^C NMR (100 MHz, CDCl_3_) δ 80.5 (d, *J* = 61.8 Hz), 80.8 (d, *J* = 52.9 Hz), 116.53, 116.55, 125.2, 130.1, 152.3 (d, *J* = 288.1 Hz), 152.6 (d, *J* = 283.0 Hz), 153.9; ^19^F NMR (376 MHz, CDCl_3_) δ −80.5 (s, 1F), −80.6 (s, 1F); EIMS *m/z*: 250, 252 [M]^+^; HREIMS: [M]^+^ calcd for C_8_H_5_BrClFO, 249.9196, 251.9176; found, 249.9202, 251.9172.

**(3-Chloro-4-fluoro-4-phenoxybut-3-en-1-yn-1-yl)trimethylsilane (7):** To a solution of **2a** (0.5 mmol), bis(triphenylphosphine)palladium dichloride (4 mol %), copper iodide (4 mol %) and triethylamine (0.75 mmol) in THF (2.5 mL) was added dropwise trimethylsislylacetylene (1.0 mmol) for 1 min at room temperature. The solution was stirred at rt until the Sonogashira coupling reaction was completed. The reaction mixture was filtered and concentrated under reduced pressure. The residue was purified by column chromatography (hexane only) to afford **7** in 80% yield (107.2 mg) as a yellow oil. ^1^H NMR (400 MHz, CDCl_3_) δ 0.127 and 0.239 (s, 9H), 7.06–7.14 (m, 2H), 7.16–7.22 (m, 1H), 7.32–7.41 (m, 2H); ^13^C NMR (100 MHz, CDCl_3_) δ −0.364, −0.259, 85.3 (d, *J* = 45.2 Hz), 85.6 (d, *J* = 53.5 Hz), 94.3 (d, *J* = 6.8 Hz), 94.8 (d, *J* = 2.7 Hz), 103.4 (d, *J* = 5.4 Hz), 104.1 (d, *J* = 8.1 Hz), 117.2, 117.3, 125.2, 125.3 130.0, 130.1, 153.6 (d, *J* = 1.9 Hz), 154.4 (d, *J* = 3.0 Hz), 158.1 (d, *J* = 292.6 Hz), 158.7 (d, *J* = 290.7 Hz); ^19^F NMR (376 MHz, CDCl_3_) δ −73.3 (s, 1F), −78.4 (s, 1F); EIMS *m/z*: 268, 270 [M]^+^; HREIMS: [M]^+^ calcd for C_13_H_14_ClFSi, 268.0486, 270.0457; found, 268.0490, 270.0452.

## Supporting Information

File 1 Characterization data for **2b**–**p** and copies of ^1^H, ^13^C, and ^19^F NMR spectra.
