# Supplementary material for: Simple synthesis of multi-halogenated alkenes from 2-bromo-2-chloro-1,1,1-trifluoroethane (halothane)
Source: Beilstein J Org Chem. 2022 Nov 21;18:1567–74. doi: 10.3762/bjoc.18.167 (PMC9704012; doi:10.3762/bjoc.18.167)

BEILSTEIN JOURNAL OF ORGANIC CHEMISTRY

# Supporting Information

for

# Simple synthesis of multi-halogenated alkenes from 2-bromo-2-chloro-1,1,1-trifluoroethane (halothane)

Yukiko Karuo, Atsushi Tarui, Kazuyuki Sato, Kentaro Kawai and Masaaki Omote

*Beilstein J. Org. Chem.* **2022,** *18,* 1567–1574. doi:10.3762/bjoc.18.167

## Characterization data for 2b–p and copies of $^{1}H$, $^{13}C$, and $^{19}F$ NMR spectra

License and Terms: This is a supporting information file under the terms of the Creative Commons Attribution License (https://creativecommons.org/licenses/by/4.0). Please note that the reuse, redistribution and reproduction in particular requires that the author(s) and source are credited and that individual graphics may be subject to special legal provisions.
The license is subject to the *Beilstein Journal of Organic Chemistry* terms and conditions: (https://www.beilstein-journals.org/bjoc/terms)

Experimental section ……………………...……………………………. S2
Spectroscopic data ……….…………………….…………….……………..S2

$^{1}$H, $^{13}$C and $^{19}$F NMR charts…………………………………..…........ S9−S34

**Spectroscopic data:**

**2-Bromo-2-chloro-1-fluoroethenyl 1-naphthyl ether (2b)**

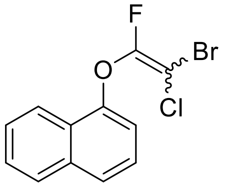


Reaction condition: **3b** (1.05 equiv) was used.

The title product (**2b**) was purified by column chromatography and preparative TLC (hexane only). **2b** was obtained in 70% yield (209.9 mg).

A pale yellow oil.; $^{1}$H NMR (400 MHz, $CDCl_3$) δ: 7.09-7.15 (1H, m), 7.43 (1H, dd, $J$ = 7.9, 7.9 Hz), 7.53-7.61 (2H, m), 7.69 (1H, d, $J$ = 8.3 Hz), 7.84-7.92 (1H, m), 8.17-8.26 (1H, m); $^{13}$C NMR (100 MHz, $CDCl_3$) δ: 80.7 (d, $J$ = 60.6 Hz), 81.0 (d, $J$ = 52.1 Hz), 109.6, 109.7, 121.38, 121.41, 124.9, 125.01, 125.03, 125.4, 126.7, 127.2, 127.9, 134.9, 149.6 (d, $J$ = 3.8 Hz), 149.7 (d, $J$ = 3.8 Hz), 152.5 (d, $J$ = 287.6 Hz), 152.9 (d, $J$ = 282.7 Hz); $^{19}$F NMR (376 MHz, $CDCl_3$) δ: -80.70 (1F, d, $J$ = 2.2 Hz), -80.72 (1F, d, $J$ = 2.2 Hz); MS (EI) *m/z*: 300, 302 ($M^+$); HRMS (EI) Calcd. for $C_{12}H_7BrClFO$: 299.9353, 301.9332 ($M^+$), Found: 299.9350, 301.9330.

**2-Bromo-2-chloro-1-fluoroetheyl *o*-phenylphenyl ether (2c)**

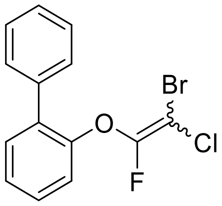


Reaction condition: **3c** (1.05 equiv) was used.

The title product (**2c**) was purified by column chromatography and preparative TLC (hexane only). **2c** was obtained in 85% yield (278.9 mg).

A pale yellow oil; $^{1}$H NMR (400 MHz, $CDCl_3$) δ: 7.09-7.16 (1H, m), 7.27 (1H, t, $J$ = 7.5 Hz), 7.33-7.47 (5H, m), 7.48-7.55 (2H, m); $^{13}$C NMR (100 MHz, $CDCl_3$) δ: 80.1 (d, $J$ = 60.8 Hz), 80.5 (d, $J$ = 51.8 Hz), 115.5, 115.6, 125.4, 127.8, 128.4, 128.9, 129.5, 129.6, 131.9, 132.1, 136.7, 150.4, 152.0 (d, $J$ = 287.9 Hz), 152.5 (d, $J$ = 282.6 Hz); $^{19}$F NMR (376 MHz, $CDCl_3$) δ: -80.8 (1F, d, $J$ = 2.1 Hz), -80.9 (1F, d, $J$ = 2.1 Hz); MS (EI) *m/z*: 326, 328 ($M^+$); HRMS (EI) Calcd. for $C_{14}H_9BrClFO$: 325.9509, 327.9489 ($M^+$), Found: 325.9510, 327.9485.

**2-Bromo-2-chloro-1-fluoroethenyl *m*-phenylphenyl ether (2d)**

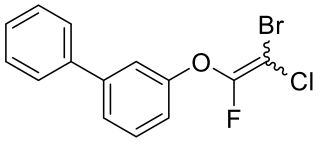

The title product (**2d**) was purified by column chromatography and preparative TLC (hexane only). **2d** was obtained in 68% yield (221.8 mg).

A colorless oil; $^{1}$H NMR (400 MHz, $CDCl_3$) δ: 7.07 (1H, d, *J* = 7.3 Hz), 7.29 (1H, s), 7.35-7.52 (5H, m), 7.58 (2H, d, *J* = 7.8 Hz); $^{13}$C NMR (100 MHz, $CDCl_3$) δ: 80.7 (d, *J* = 58.7 Hz), 80.9 (d, *J* = 52.9 Hz), 115.1, 115.3, 124.0, 127.3, 128.1, 129.1, 130.4, 140.0, 143.7, 152.2 (d, *J* = 287.7 Hz), 152.7 (d, *J* = 283.5 Hz), 154.3; $^{19}$F NMR (376 MHz, $CDCl_3$) δ: -80.45, -80.52; MS (EI) *m/z*: 326, 328 ($M^+$); HRMS (EI) Calcd. for $C_{14}H_9BrClFO$: 325.9509, 327.9489 ($M^+$), Found: 325.9507, 327.9489.

**2-Bromo-2-chloro-1-fluoroethenyl *p*-phenylphenyl ether (2e)**

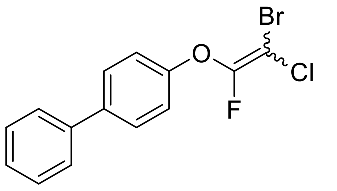


The title product (**2e**) was purified by column chromatography and preparative TLC (hexane only). **2e** was obtained in 63% yield (205.2 mg).

A white solid; $^{1}$H NMR (400 MHz, $CDCl_3$) δ: 7.16 (2H, d, *J* = 8.2 Hz), 7.36 (1H, tt, *J* = 7.3, 1.1 Hz), 7.44 (2H, dd, *J* = 7.3, 7.3 Hz), 7.55 (2H, d, *J* = 7.3 Hz), 7.59 (2H, d, *J* = 8.2 Hz); $^{13}$C NMR (100 MHz, $CDCl_3$) δ: 80.7 (d, *J* = 61.1 Hz), 80.9 (d, *J* = 52.7 Hz), 116.82, 116.84, 127.2, 127.6, 128.8, 129.0, 138.5, 140.1, 152.2 (d, *J* = 287.8 Hz), 152.6 (d, *J* = 282.4 Hz), 153.3; $^{19}$F NMR (376 MHz, $CDCl_3$) δ: -80.56 (1F, s), -80.61 (1F, s); MS (EI) *m/z*: 326, 328 ($M^+$); HRMS (EI) Calcd. for $C_{14}H_9BrClFO$: 325.9509, 327.9489 ($M^+$), Found: 325.9501, 327.9488.

**2-Bromo-2-chloro-1-fluoroethenyl *p*-methoxyphenyl ether (2f)**

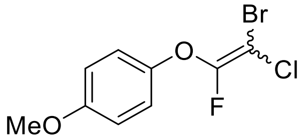


Reaction condition: **3f** (1.05 mmol) was used.

The title product (**2f**) was purified by column chromatography (hexane only to hexane/AcOEt = 12:1) and was obtained in 76% yield (213.9 mg).

A yellow oil; $^{1}$H NMR (400 MHz, $CDCl_3$) δ: 3.80 (3H, s), 6.88 (2H, d, *J* = 8.6 Hz), 7.04 (2H, d, *J* = 8.6 Hz); $^{13}$C NMR (100 MHz, $CDCl_3$) δ: 55.8, 79.6 (d, *J* = 62.3 Hz), 79.8 (d, *J* = 53.4 Hz), 115.0, 118.0, 118.1, 147.59, 147.62, 152.8 (d, *J* = 287.8 Hz), 153.2 (d, *J* = 282.7 Hz), 157.0; $^{19}$F NMR (376 MHz, $CDCl_3$) δ: -80.58 (1F, s), -80.60 (1F, s); MS (EI) *m/z*: 280, 282 ($M^+$); HRMS (EI) Calcd. for $C_9H_7BrClFO_2$: 279.9302, 281.9282 ($M^+$), Found: 279.9307, 281.9278.

**2-Bromo-2-chloro-1-fluoroethenyl *o-tert*-butylphenyl ether (2g)**

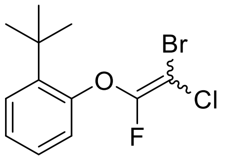


Reaction condition: **3g** (1.05 mmol) was used.
The title product (**2g**) was purified by column chromatography and preparative TLC (hexane only). **2g** was obtained in 43% yield (131.0 mg).
A colorless oil; $^1$H NMR (400 MHz, $CDCl_3$) δ: 1.41 (9H, s), 6.91-6.98 (1H, m), 7.12 (1H, ddd, $J$ = 7.6, 7.6, 2.3 Hz), 7.22 (1H, ddd, $J$ = 7.6, 7.6, 2.7 Hz), 7.39 (1H, dd, $J$ = 7.6, 2.3 Hz); $^{13}$C NMR (100 MHz, $CDCl_3$) δ: 30.1, 34.9, 80.4 (d, $J$ = 60.9 Hz), 80.8 (d, $J$ = 52.3 Hz), 114.8, 114.9, 124.79, 124.81, 127.5, 127.9, 138.8, 151.5 (d, $J$ = 288.5 Hz), 151.9 (d, $J$ = 282.8Hz), 152.27 (d, $J$ = 2.8 Hz), 152.33 (d, $J$ = 2.8 Hz); $^{19}$F NMR (376 MHz, $CDCl_3$) δ: -81.4 (1F, d, $J$ = 2.7 Hz), -81.7 (1F, d, $J$ = 2.7 Hz); MS (EI) *m/z*: 306, 308 ($M^+$); HRMS (EI) Calcd. for $C_{12}H_{13}BrClFO$: 305.9822, 307.9802 ($M^+$), Found: 305.9825, 307.9792.

**2-Bromo-2-chloro-1-fluoroethenyl *m*-trifluoromethylphenyl ether (2h)**

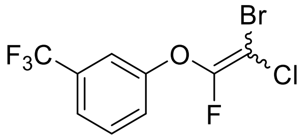


Reaction condition: **3h** (1.05 mmol) and DME (2.5 mL) was used.
The title product (**2h**) was purified by column chromatography and preparative TLC (hexane only). **2h** was obtained in 39% yield (125.4 mg).
A colorless oil; $^1$H NMR (400 MHz, $CDCl_3$) δ: 7.27 (1H, d, $J$ = 7.9 Hz), 7.34 (1H, s), 7.48 (1H, d, $J$ = 7.6 Hz), 7.53 (1H, dd, $J$ = 7.9, 7.6 Hz); $^{13}$C NMR (100 MHz, $CDCl_3$) δ: 81.5 (d, $J$ = 59.8 Hz), 81.8 (d, $J$ = 51.0 Hz), 113.9, 119.7, 122.0, 123.4 (q, $J$ = 272.6 Hz), 130.9, 132.9 (q, $J$ = 33.3 Hz), 151.7 (d, $J$ = 288.2 Hz), 152.1 (d, $J$ = 283.4 Hz), 153.82 (d, $J$ = 3.6 Hz), 153.85 (d, $J$ = 3.6 Hz); $^{19}$F NMR (376 MHz, $CDCl_3$) δ: -62.7 (3F, s), -81.4 (1F, s), -81.5 (1F, s); MS (EI) *m/z*: 318 ($M^+$); HRMS (EI) Calcd. for $C_9H_4BrClF_4O$: 317.9070 ($M^+$), Found: 317.9064.

**2-Bromo-2-chloro-1-fluoroethenyl *p*-nitrophenyl ether (2i)**

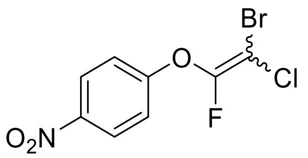


Reaction condition: **3i** (1.05 mmol) and DME (2.5 mL) was used.
The title product (**2i**) was purified by column chromatography and preparative TLC (hexane only). **2i**

was obtained in 18% yield (54.0 mg).

A pale yellow oil; $^{1}$H NMR (400 MHz, $CDCl_3$) δ: 7.21 (2H, d, *J* = 8.5 Hz), 8.30 (2H, d, *J* = 8.5 Hz); $^{13}$C NMR (100 MHz, $CDCl_3$) δ: 82.5 (d, *J* = 58.1 Hz), 82.9 (d, *J* = 49.6 Hz), 116.7, 126.3, 144.9, 151.2 (d, *J* = 289.1 Hz), 151.5 (d, *J* = 283.6 Hz), 157.96 (d, *J* = 4.1 Hz), 158.03 (d, *J* = 4.1 Hz); $^{19}$F NMR (376 MHz, $CDCl_3$) δ: -81.6 (1F, s), -81.8 (1F, s); MS (EI) *m/z*: 295, 297 ($M^+$); HRMS (EI) Calcd. for $C_8H_4BrClFNO_3$: 294.9047, 296.9027 ($M^+$), Found: 294.9055, 296.9023.

**<u>*o*-Allylphenyl 2-bromo-2-chloro-1-fluoroethenyl ether (2j)</u>**

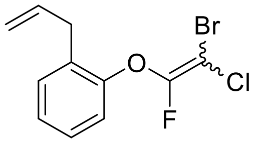


Reaction condition: **3j** (1.05 eq.) was used.

The title product (**2j**) was purified by column chromatography and preparative TLC (hexane only). **2j** was obtained in 75% yield (217.8 mg).

A colorless oil; $^{1}$H NMR (400 MHz, $CDCl_3$) δ: 3.45 (2H, d, *J* = 6.7 Hz), 5.03-5.12 (2H, m), 5.89-6.03 (1H, m), 6.97-7.03 (1H, m), 7.13 (1H, t, *J* = 7.3 Hz), 7.20-7.27 (2H, m); $^{13}$C NMR (100 MHz, $CDCl_3$) δ: 34.1, 80.0 (d, *J* = 61.9 Hz), 80.4 (d, *J* = 51.9 Hz), 114.8, 115.0, 116.5, 125.3, 127.8, 129.7, 131.1, 135.8, 151.7, 152.4 (d, *J* = 288.1 Hz), 152.7 (d, *J* = 282.9 Hz); $^{19}$F NMR (376 MHz, $CDCl_3$) δ: -80.3 (1F, d, *J* = 2.1 Hz), -80.4 (1F, d, *J* = 2.1 Hz); MS (EI) *m/z*: 290, 292 ($M^+$); HRMS (EI) Calcd. for $C_{11}H_9BrClFO$: 289.9509, 291.9489 ($M^+$), Found: 289.9509, 291.9489.

**<u>2-Bromo-2-chloro-1,1-fluoroethenyl *o*-propenylphenyl ether (2k)</u>**

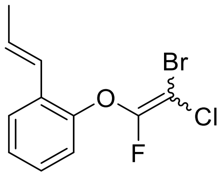


The title product (**2k**) was purified by column chromatography (hexane only) and was obtained as an inseparable mixture of *cis*-*trans* isomers (1:4) in 54% yield (157.9 mg).

A pale yellow oil; $^{1}$H NMR (400 MHz, $CDCl_3$) δ: 1.81 (ddd, *J* = 7.1, 2.2, 1.1 Hz, *cis*-isomer) and 1.92 (dd, *J* = 6.8, 1.5 Hz, *trans*-isomer) (3H), 5.92 (dq, *J* = 11.3, 7.1 Hz, *cis*-isomer) and 6.23 (dq, *J* = 15.8, 6.7 Hz, *trans*-isomer) (1H), 6.50 (d, *J* = 11.3 Hz, *cis*-isomer) and 6.68 (dq, *J* = 15.8, 1.5 Hz, *trans*-isomer) (1H), 6.99 (dq, *J* = 8.1, 1.5 Hz, *trans*-isomer) and 7.04 (dq, *J* = 8.1, 1.1 Hz, *cis*-isomer) (1H), 7.09-7.29 (2H, m), 7.33 (d, *J* = 7.6 Hz, *cis*-isomer) and 7.49 (d, *J* = 7.6 Hz, *trans*-isomer) (1H); $^{13}$C NMR (100 MHz, $CDCl_3$) δ: 14.8 (*cis*-isomer), 19.0 (*trans*-isomer), 79.6 (d, *J* = 61.4 Hz, *cis*-isomer), 79.8 (d, *J* = 61.4 Hz, *trans*-isomer), 80.0 (d, *J* = 52.9 Hz, *cis*-isomer), 80.2 (d, *J* = 52.4 Hz, *trans*-isomer), 115.4, 115.6, 123.5 (*cis*-isomer), 123.6 (*cis*-isomer), 123.9 (*trans*-isomer), 124.0 (*trans*-isomer), 124.8 (*cis*-isomer), 125.3 (*trans*-isomer), 127.2 (*trans*-isomer), 127.78 (*cis*-isomer), 127.80 (*cis*-isomer), 128.0 (*trans*-isomer), 128.3 (*cis*-isomer), 128.38 (*trans*-isomer), 128.40 (*trans*-isomer),

128.86 (*trans*-isomer), 128.89 (*trans*-isomer), 129.28 (*cis*-isomer), 129.32 (*cis*-isomer), 131.1 (*cis*-isomer), 150.3 (*trans*-isomer), 151.1 (*cis*-isomer), 152.4 (d, *J* = 287.2 Hz), 152.8 (d, *J* = 282.3 Hz); $^{19}$F NMR (376 MHz, $CDCl_3$) δ: -80.21 (1F, s, *cis*-isomer), -80.22 (1F, s, *cis*-isomer), -80.22 (1F, d, *J* = 1.5 Hz, *trans*-isomer), -80.23 (1F, d, *J* = 1.5 Hz, *trans*-isomer); MS (EI) *m*/*z*: 290, 292 ($M^+$); HRMS (EI) Calcd. for $C_{11}H_9BrClFO$: 289.9509, 291.9489 ($M^+$), Found: 289.9505, 291.9493.

**(*E*)-2'-(2-Bromo-2-chloro-1-fluoroethenyloxy)-chalcone (2l)**

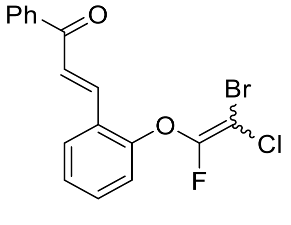


Reaction condition: **3l** (1.05 equiv) was used.

The title product (**2l**) was purified by column chromatography (hexane/AcOEt = 14:1) and was obtained in 51% yield (191.4 mg).

An orange oil; $^1$H NMR (400 MHz, $CDCl_3$) δ: 7.07-7.13 (1H, m), 7.26 (1H, dd, *J* = 7.5, 7.5 Hz), 7.40-7.47 (1H, m), 7.52 (2H, dd, *J* = 7.5, 7.5 Hz), 7.56-7.64 (2H, m), 7.52 (1H, dd, *J* = 7.8, 1.5 Hz), 8.00-8.04 (2H, m), 8.07 (1H, d, *J* = 16.0 Hz); $^{13}$C NMR (100 MHz, $CDCl_3$) δ: 81.2 (d, *J* = 60.0 Hz), 81.6 (d, *J* = 51.1 Hz), 115.4, 115.5, 124.8, 125.3, 125.4, 128.75, 128.83, 129.1, 129.2, 131.8, 133.1, 137.75, 137.79, 138.1, 151.9 (d, *J* = 288.1 Hz), 152.1 (d, *J* = 282.7 Hz), 152.5 (d, *J* = 3.3 Hz), 152.6 (d, *J* = 3.7 Hz), 190.6; $^{19}$F NMR (376 MHz, $CDCl_3$) δ: -80.5 (1F, d, *J* = 2.4 Hz), -80.6 (1F, d, *J* = 2.3 Hz); MS (EI) *m*/*z*: 380 ($M^+$); HRMS (EI) Calcd. for $C_{17}H_{11}BrClFO_2$: 379.9615 ($M^+$), Found: 379.9615.

**2-Bromo-2-chloro-1-fluoroethenyl *p*-formylphenyl ether (2m)**

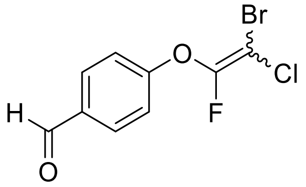


Reaction condition: **3m** (1.05 mmol) was used.

The title product (**2m**) was purified by column chromatography and preparative TLC (hexane/AcOEt = 14:1). **2m** was obtained in 9% yield (25.7 mg).

A yellow oil; $^1$H NMR (400 MHz, $CDCl_3$) δ: 7.23 (2H, d, *J* = 8.4 Hz), 7.94 (2H, d, *J* = 8.5 Hz), 9.99 (1H, s); $^{13}$C NMR (100 MHz, $CDCl_3$) δ: 82.0 (d, *J* = 59.5 Hz), 82.4 (d, *J* = 51.0 Hz), 116.7, 132.2, 133.5, 151.4 (d, *J* = 288.6 Hz), 151.8 (d, *J* = 283.3 Hz), 158.06 (d, *J* = 5.0 Hz), 158.11 (d, *J* = 5.0 Hz), 190.5; $^{19}$F NMR (376 MHz, $CDCl_3$) δ: -81.1 (1F, s), -81.3 (1F, s); MS (EI) *m*/*z*: 278, 280 ($M^+$); HRMS (EI) Calcd. for $C_9H_5BrClFO_2$: 277.9145, 279.9125 ($M^+$), Found: 277.9141, 279.9125.

**2-Bromo-2-chloro-1-fluoroethenyl *p*-ethoxycarbonylphenyl ether (2n)**

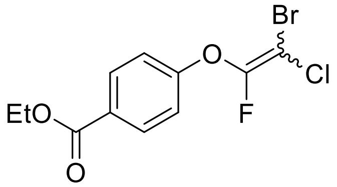


Reaction condition: **3n** (1.05 mmol) and THF was used.

The title product (**2n**) was purified by column chromatography (hexane/AcOEt = 19:1) and preparative TLC (hexane/AcOEt = 29:1). **2n** was obtained in 32% yield (102.7 mg).

A yellow oil; $^{1}$H NMR (400 MHz, $CDCl_3$) δ: 1.39 (3H, t, $J$ = 7.0 Hz), 4.38 (2H, q, $J$ = 7.0 Hz), 7.12 (2H, d, $J$ = 8.5 Hz), 8.08 (2H, d, $J$ = 8.5 Hz); $^{13}$C NMR (100 MHz, $CDCl_3$) δ: 14.5, 61.3, 81.6 (d, $J$ = 59.9 Hz), 81.9 (d, $J$ = 51.2 Hz), 116.0, 127.5, 132.0, 151.7 (d, $J$ = 288.0 Hz), 152.0 (d, $J$ = 283.2 Hz), 157.0 (d, $J$ = 4.3 Hz), 157.1 (d, $J$ = 4.3 Hz), 165.7; $^{19}$F NMR (376 MHz, $CDCl_3$) δ: -80.9 (1F, s), -81.0 (1F, s); MS (EI) *m*/*z*: 322, 324 ($M^+$); HRMS (EI) Calcd. for $C_{11}H_9BrClFO_3$: 321.9408, 323.9387 ($M^+$), Found: 321.9401, 323.9377.

***m*-Aminophenyl 2-bromo-2-chloro-1-fluoroethenyl ether (2o)**

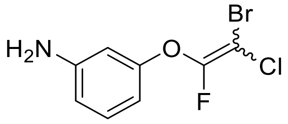


Reaction condition: **3o** (1.2 eq.) was used.

The title product (**2o**) was purified by column chromatography (hexane/AcOEt = 4:1) and was obtained in 66% yield (174.9 mg).

A yellow oil; $^{1}$H NMR (400 MHz, $CDCl_3$) δ: 3.79 (2H, br s), 6.39 (1H, s), 6.43-6.53 (2H, m), 7.12 (1H, dd, $J$ = 8.1, 8.1 Hz); $^{13}$C NMR (100 MHz, $CDCl_3$) δ: 80.4 (d, $J$ = 61.5 Hz), 80.7 (d, $J$ = 53.8 Hz), 103.02, 103.04, 106.07, 106.10, 111.8, 130.7, 148.3, 152.2 (d, $J$ = 288.1 Hz), 152.7 (d, $J$ = 282.9 Hz), 154.95, 154.98; $^{19}$F NMR (376 MHz, $CDCl_3$) δ: -80.03 (1F, s), -80.04 (1F, s); MS (EI) *m*/*z*: 265 ($M^+$); HRMS (EI) Calcd. for $C_8H_6BrClFNO$: 264.9305 ($M^+$), Found: 264.9300.

**2-Bromo-2-chloro-1-fluoroethenyl *o*-iodophenyl ether (2p)**

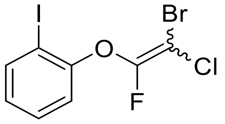


Reaction condition: Halothane (1.5 eq.) was used.

The title product (**2p**) was purified by column chromatography and preparative TLC (hexane only). **2p** was obtained in 69% yield (262.0 mg).

A yellow oil; $^{1}$H NMR (400 MHz, $CDCl_3$) δ: 6.94 (1H, ddd, $J$ = 8.0, 7.7, 1.5 Hz), 7.00-7.05 (1H, m), 7.34 (1H, ddd, $J$ = 8.0, 7.7, 1.8 Hz), 7.84 (1H, dd, $J$ = 8.0, 1.5 Hz); $^{13}$C NMR (100 MHz, $CDCl_3$) δ:

81.1 (d, *J* = 59.5 Hz), 81.5 (d, *J* = 50.5 Hz), 85.3, 115.3, 115.5, 126.8, 129.9, 140.4, 152.0 (d, *J* = 288.6 Hz), 152.4 (d, *J* = 283.1 Hz), 153.1 (d, *J* = 3.5 Hz), 153.2 (d, *J* = 3.5 Hz); $^{19}$F NMR (376 MHz, $CDCl_3$) δ: -80.8 (1F, d, *J* = 2.1 Hz), -81.0 (1F, d, *J* = 2.1 Hz); MS (EI) *m/z*: 376, 378 ($M^+$); HRMS (EI) Calcd. for $C_8H_4BrClFIO$: 375.8163, 377.8142 ($M^+$), Found: 375.8171, 377.8135.

$^{1}H$ and $^{13}C$ NMR Charts

**2-Bromo-2-chloro-1-fluoroethenyl phenyl ether (2a)**

$^{1}H$ NMR of **2a**

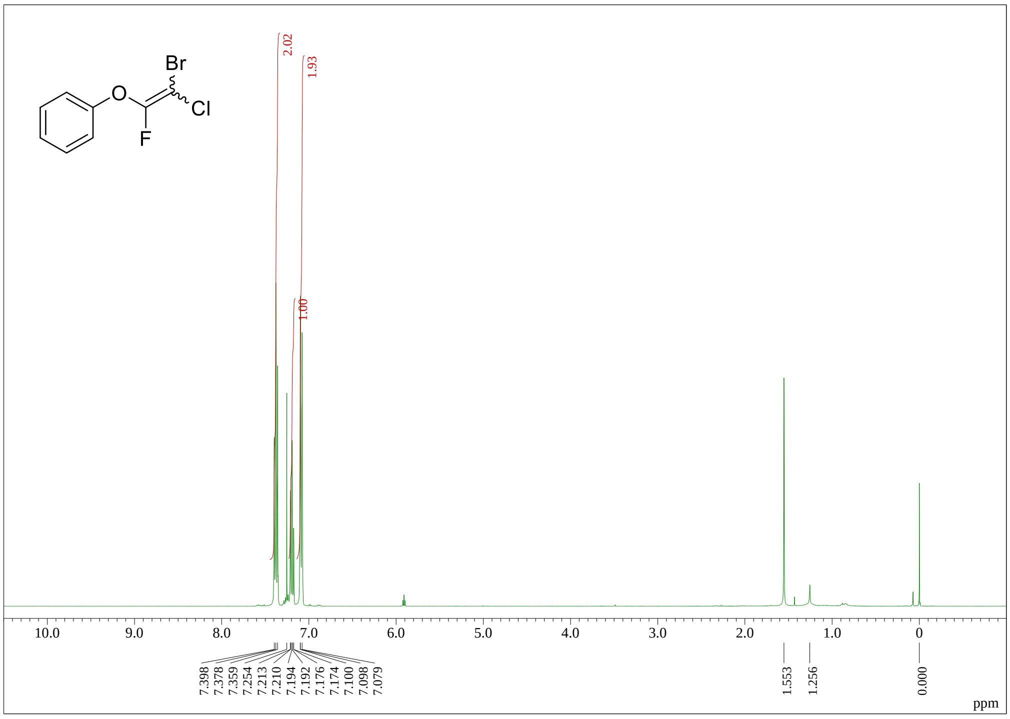


$^{13}C$ NMR of **2a**

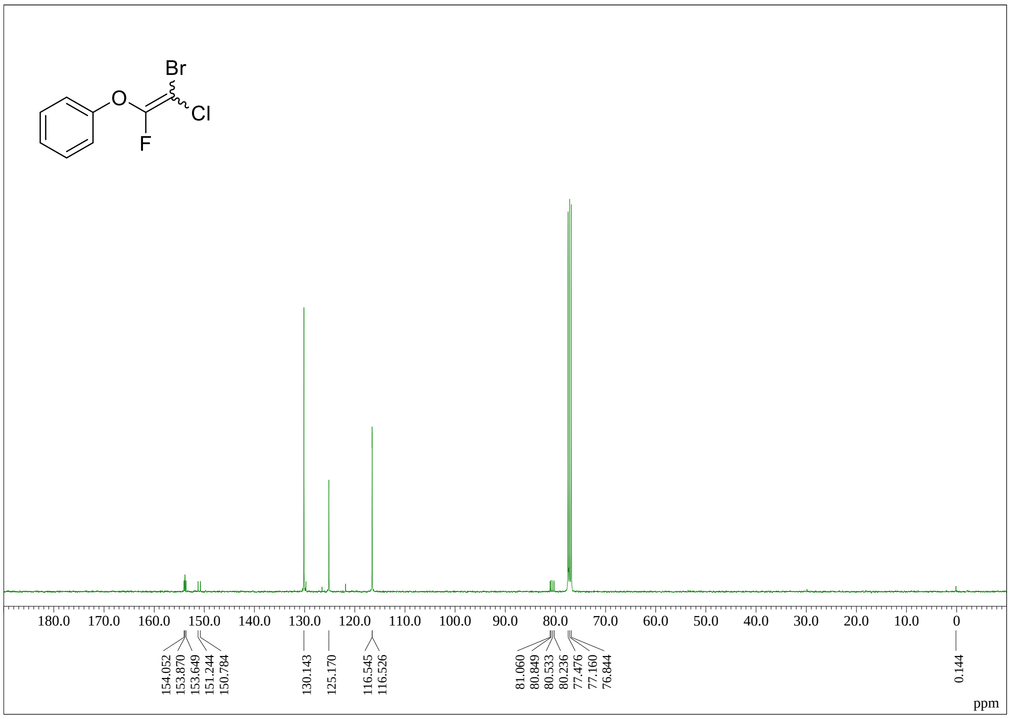

$^{19}$F NMR of **2a**

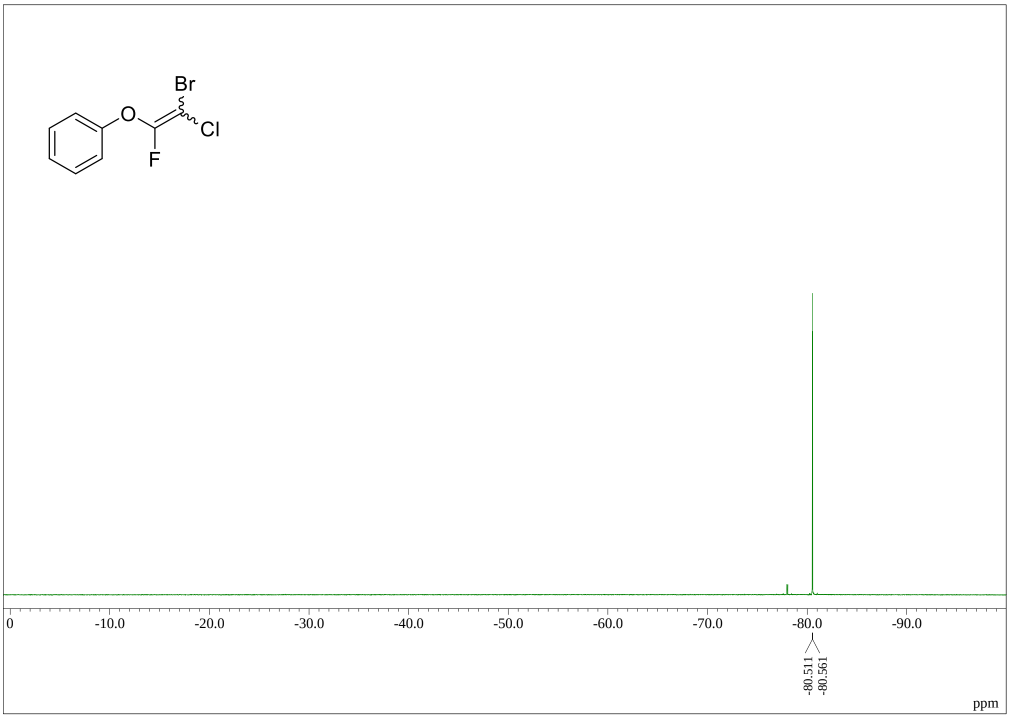


## 2-Bromo-2-chloro-1-fluoroethenyl 1-naphthyl ether (2b)

$^{1}$H NMR of **2b**

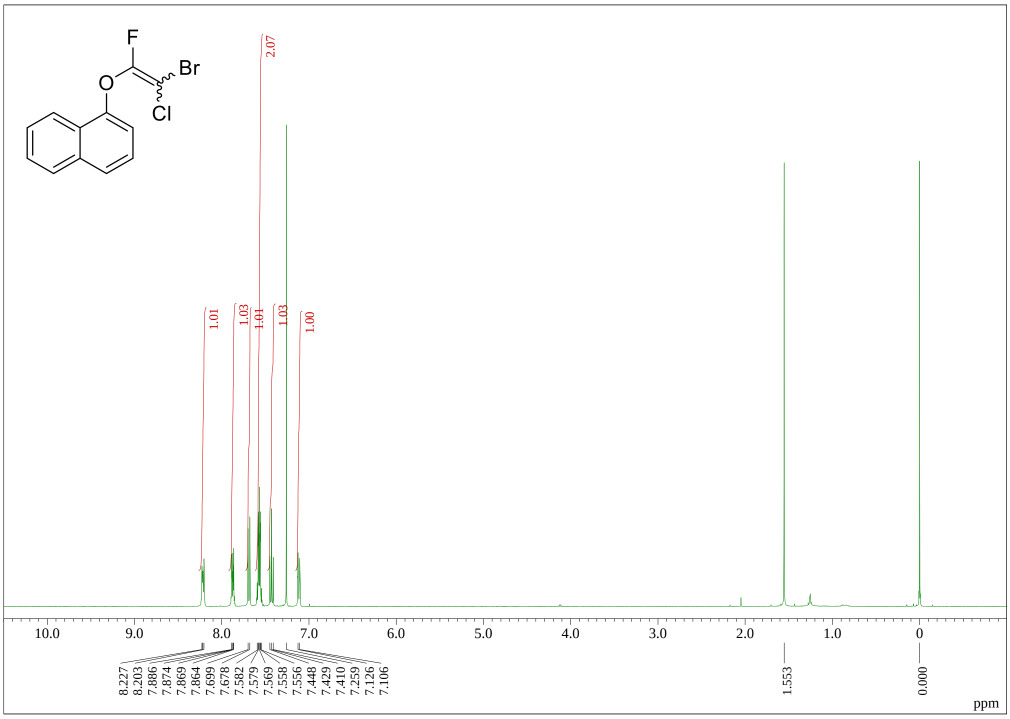

$^{13}C$ NMR of **2b**

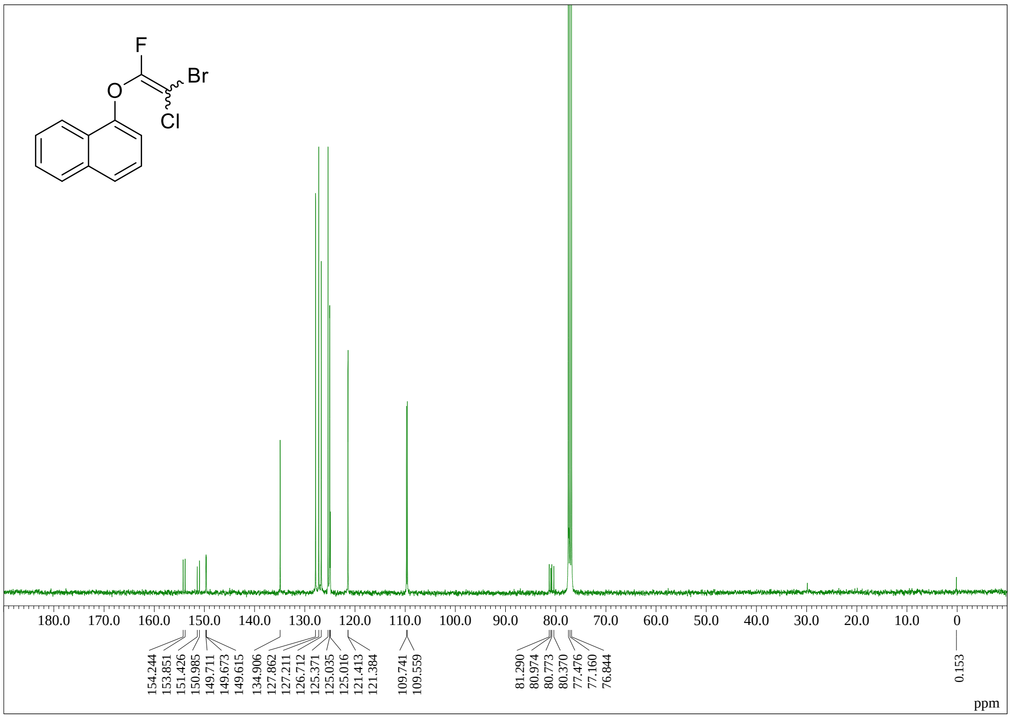


$^{19}F$ NMR of **2b**

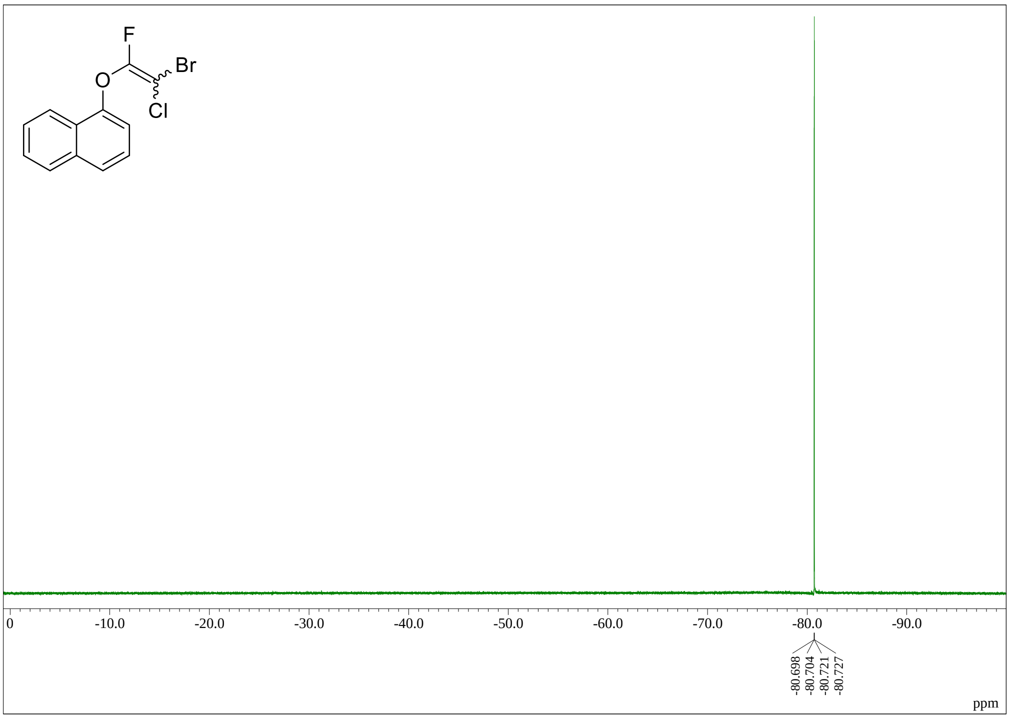

## 2-Bromo-2-chloro-1-fluoroethenyl *o*-phenylphenyl ether (2c)

$^{1}$H NMR of **2c**

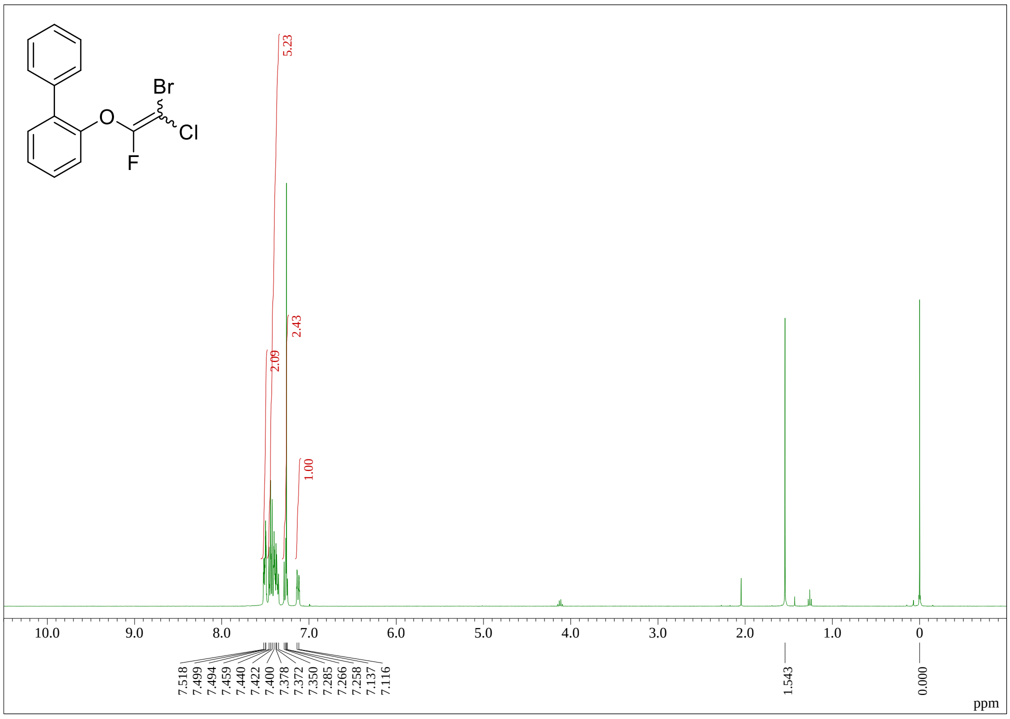

$^{13}$C NMR of **2c**

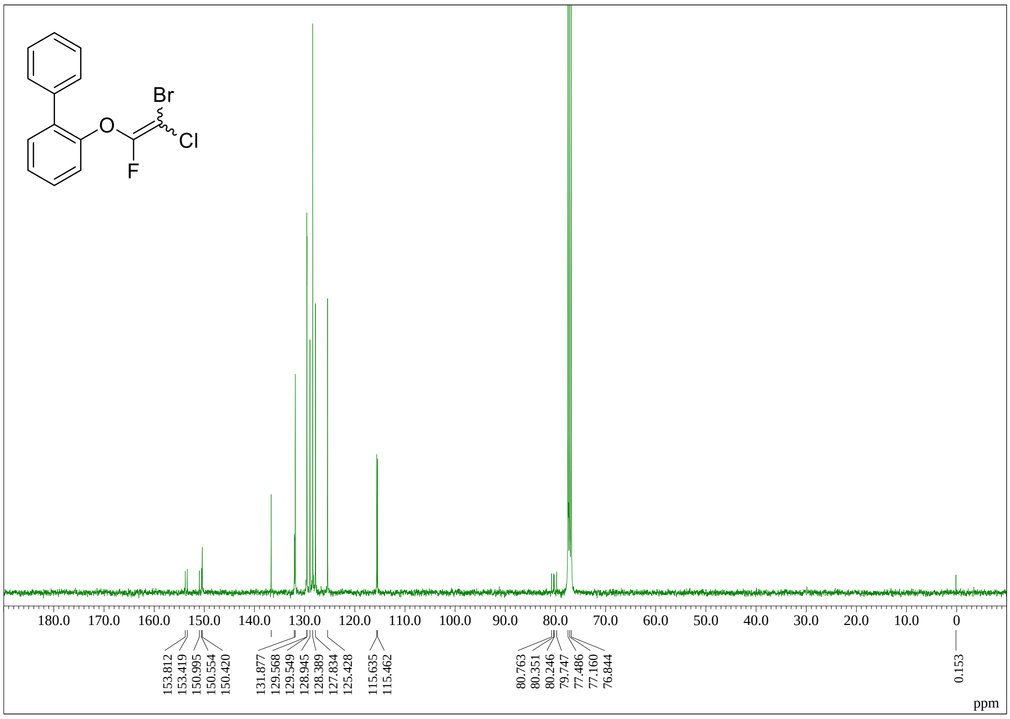

$^{19}$F NMR of **2c**

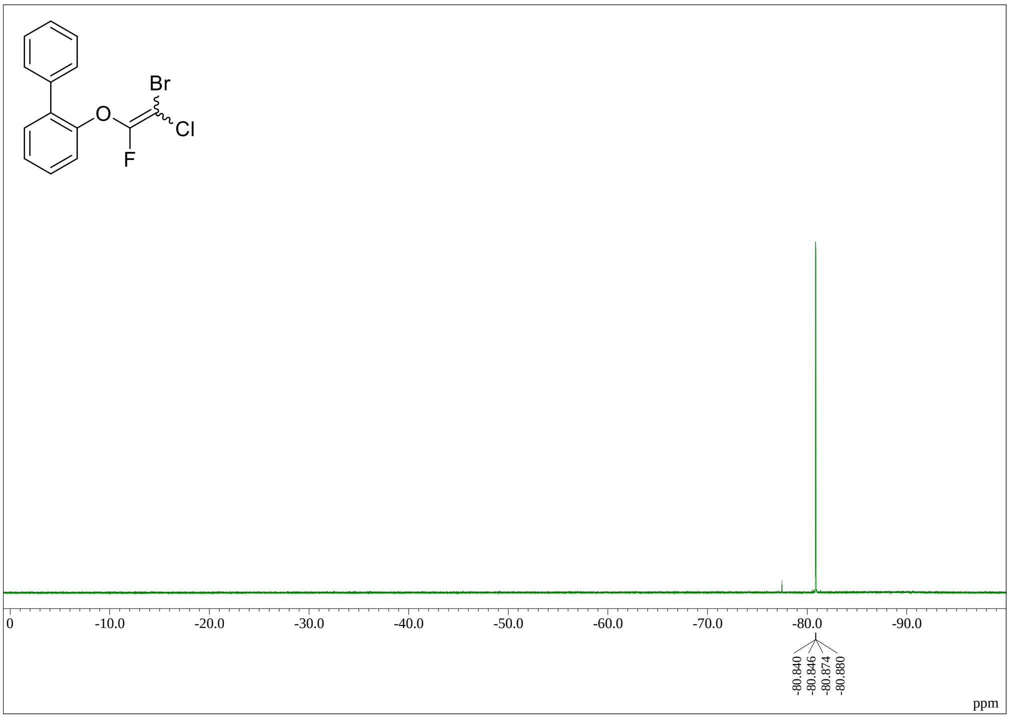


## 2-Bromo-2-chloro-1-fluoroetheyl *m*-phenylphenyl ether (2d)

$^{1}$H NMR of **2d**

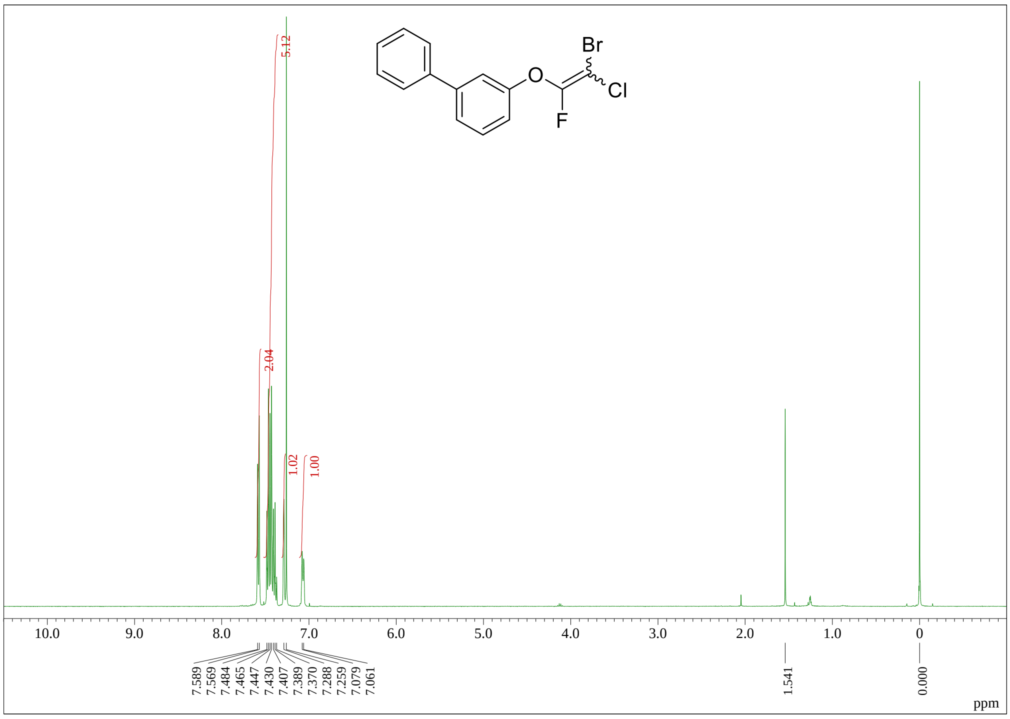

$^{13}$C NMR of **2d**

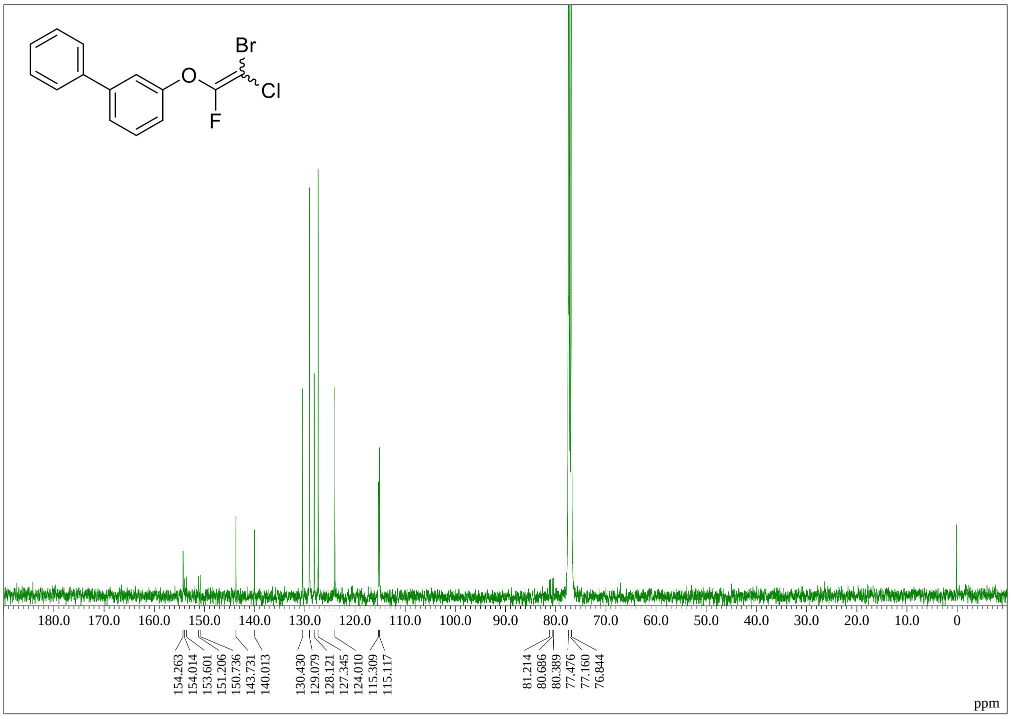


$^{19}$F NMR of **2d**

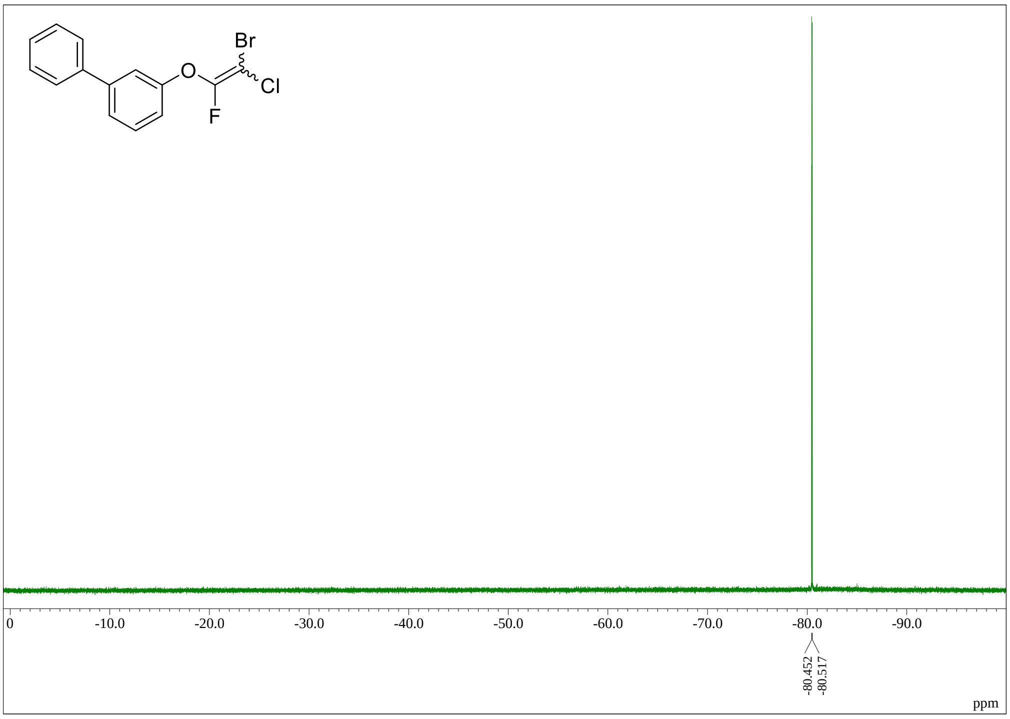

**2-Bromo-2-chloro-1-fluoroethenyl *p*-phenylphenyl ether (2e)**

$^{1}$H NMR of **2e**

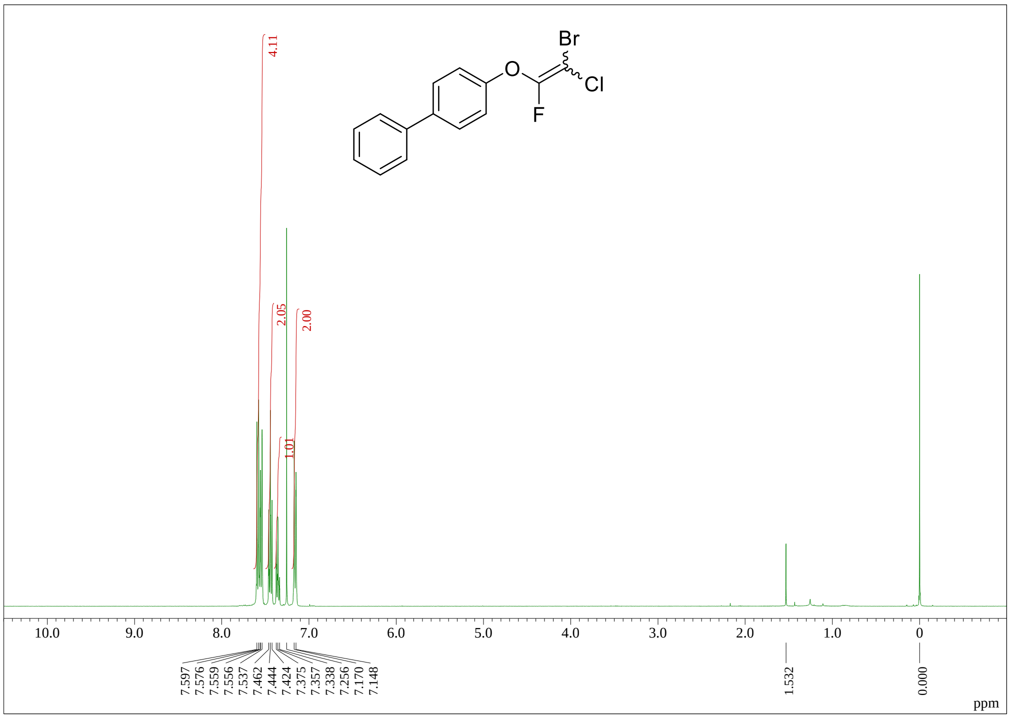


$^{13}$C NMR of **2e**

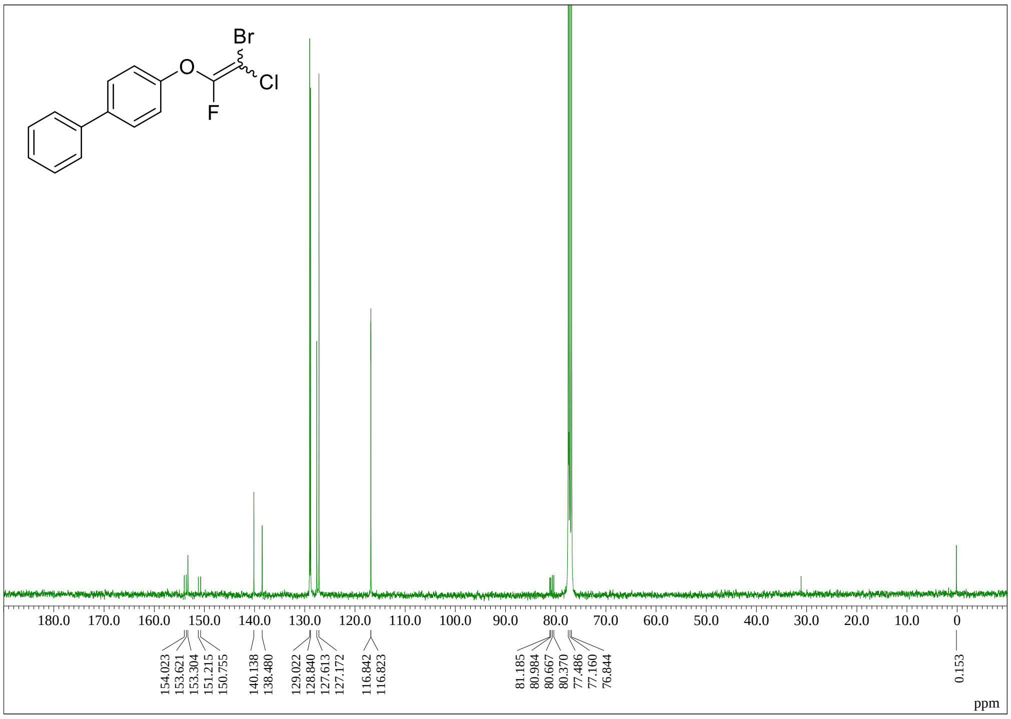

$^{19}$F NMR of **2e**

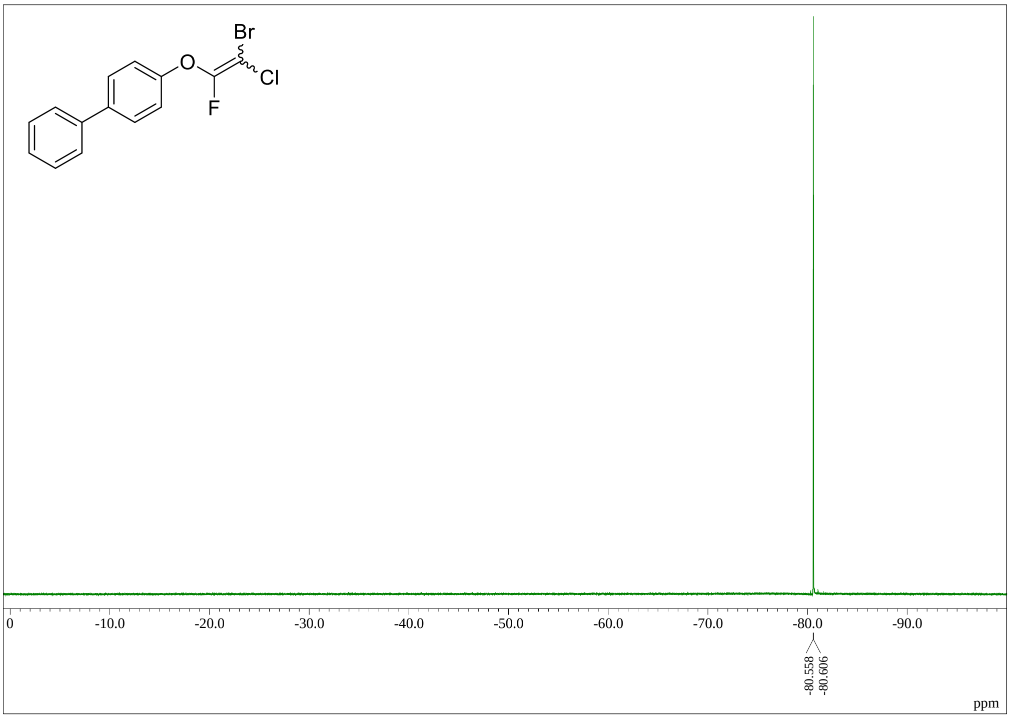


## 2-Bromo-2-chloro-1-fluoroetheyl *p*-methoxyphenyl ether (2f)

$^{1}$H NMR of **2f**

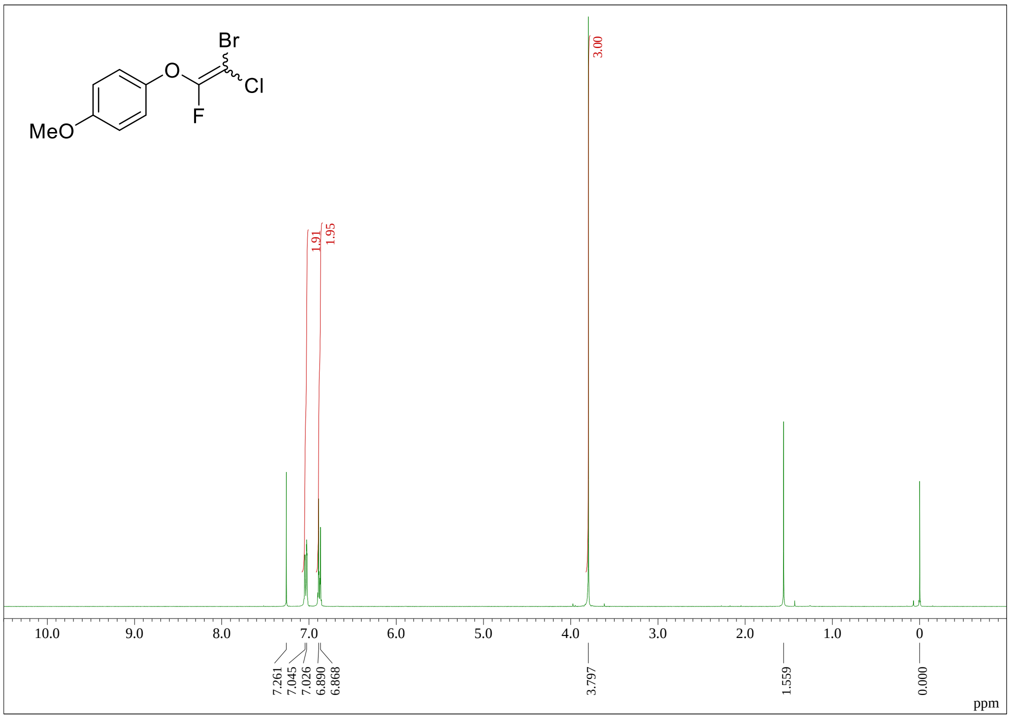

$^{13}$C NMR of **2f**

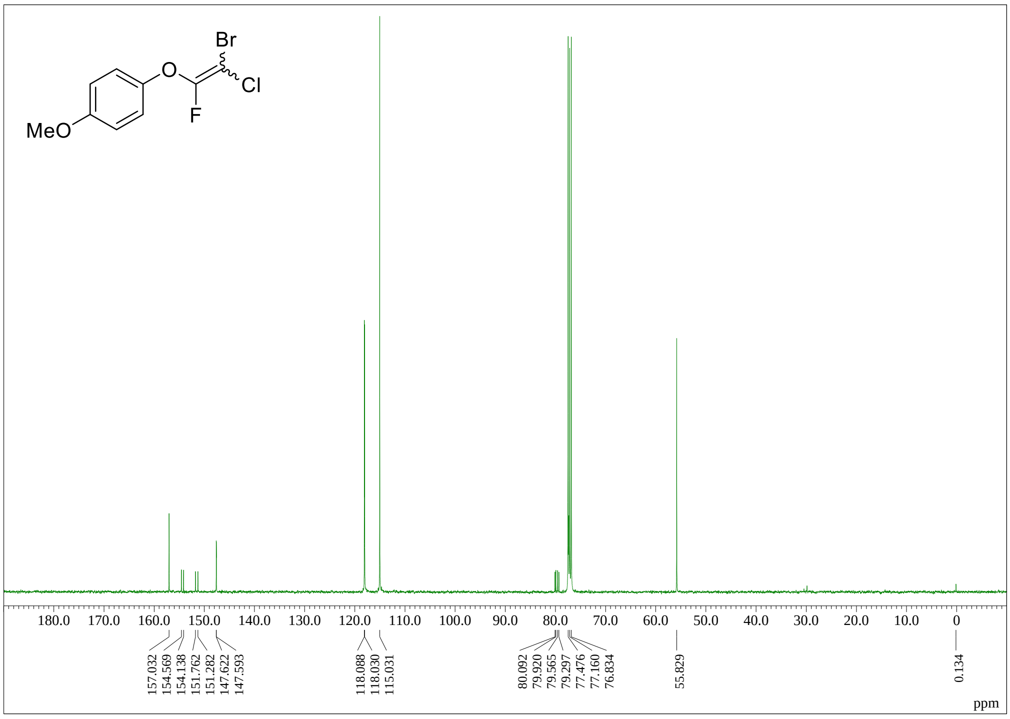


$^{19}$F NMR of **2f**

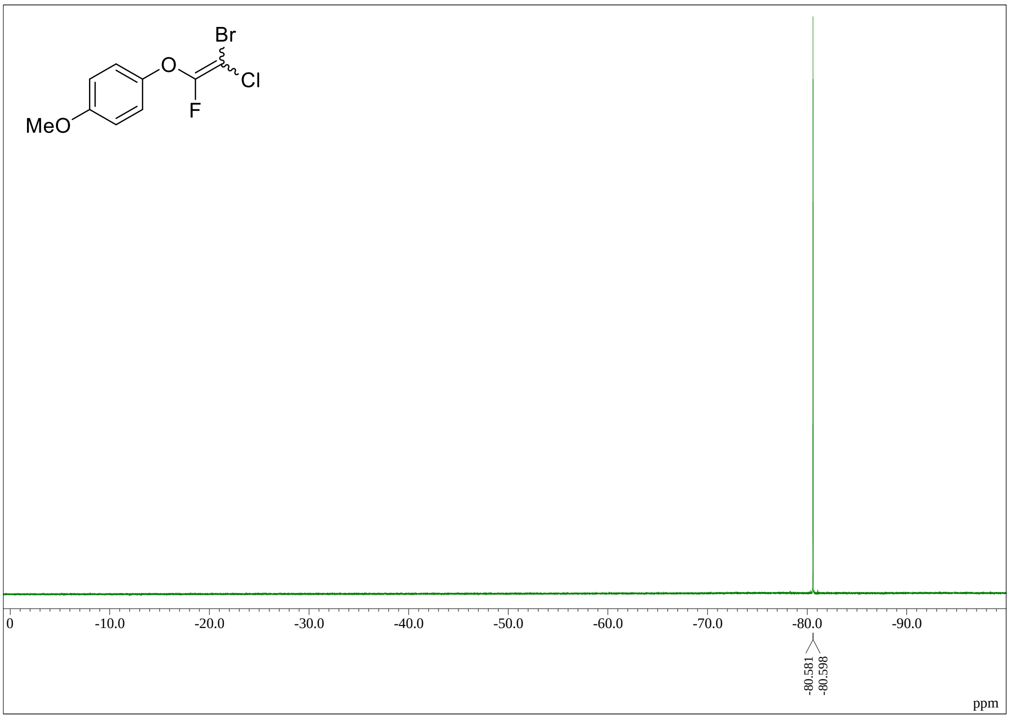

## 2-Bromo-2-chloro-1-fluoroethenyl *o-tert*-butylphenyl ether (2g)

$^1$H NMR of **2g**

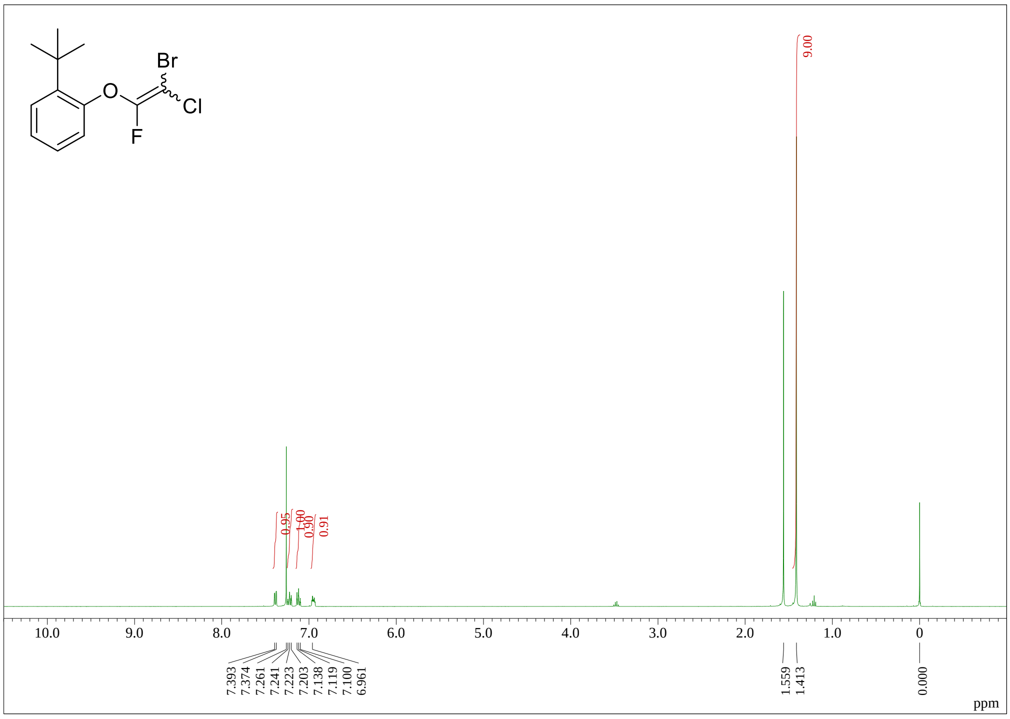


$^{13}$C NMR of **2g**

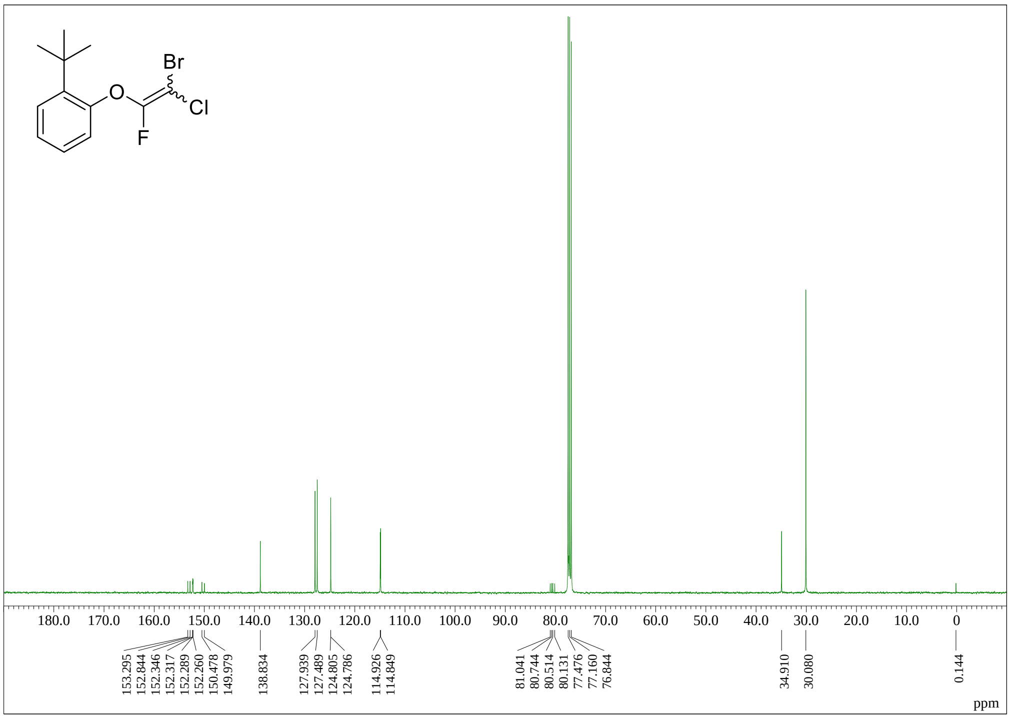

$^{19}$F NMR of **2g**

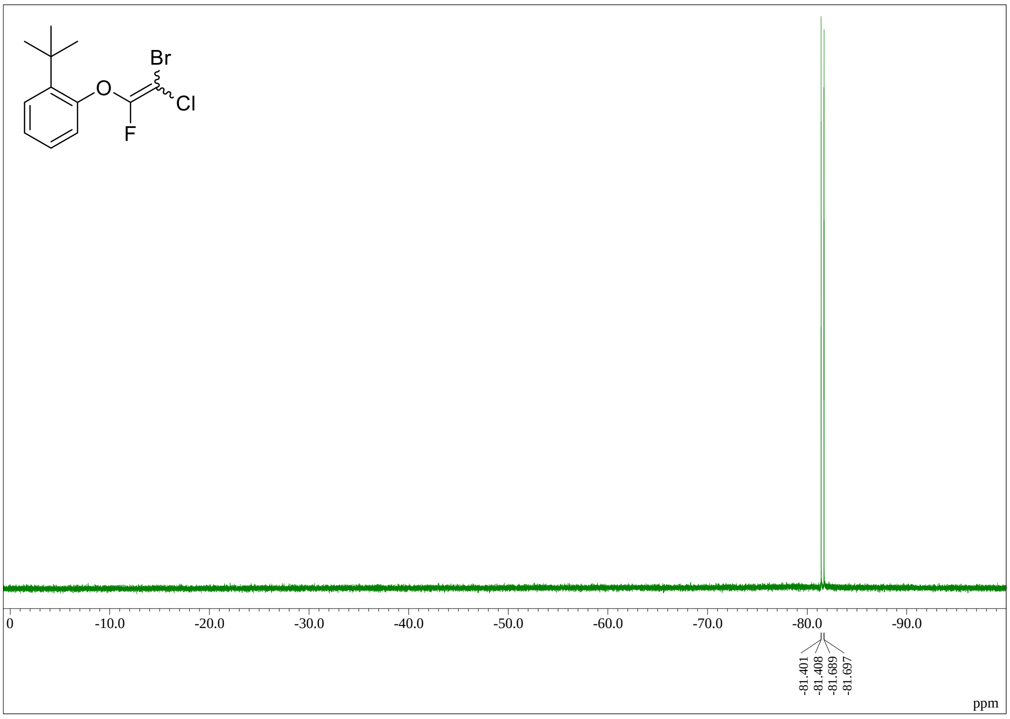


## 2-Bromo-2-chloro-1-fluoroethenyl *m*-trifluoromethylphenyl ether (2h)

$^{1}$H NMR of **2h**

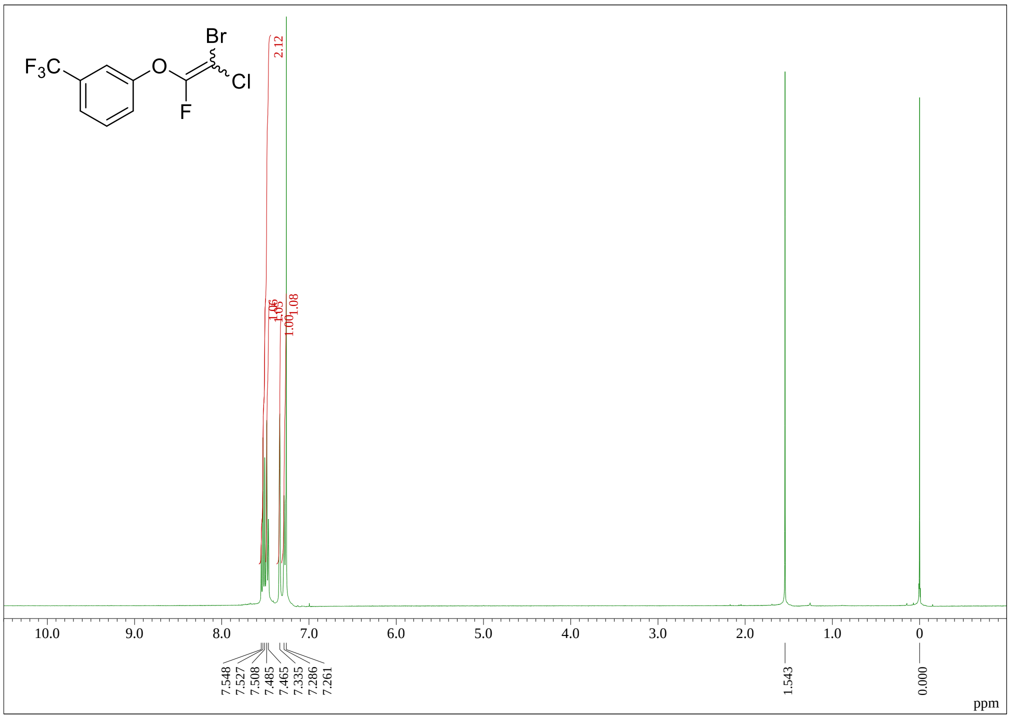

$^{13}$C NMR of **2h**

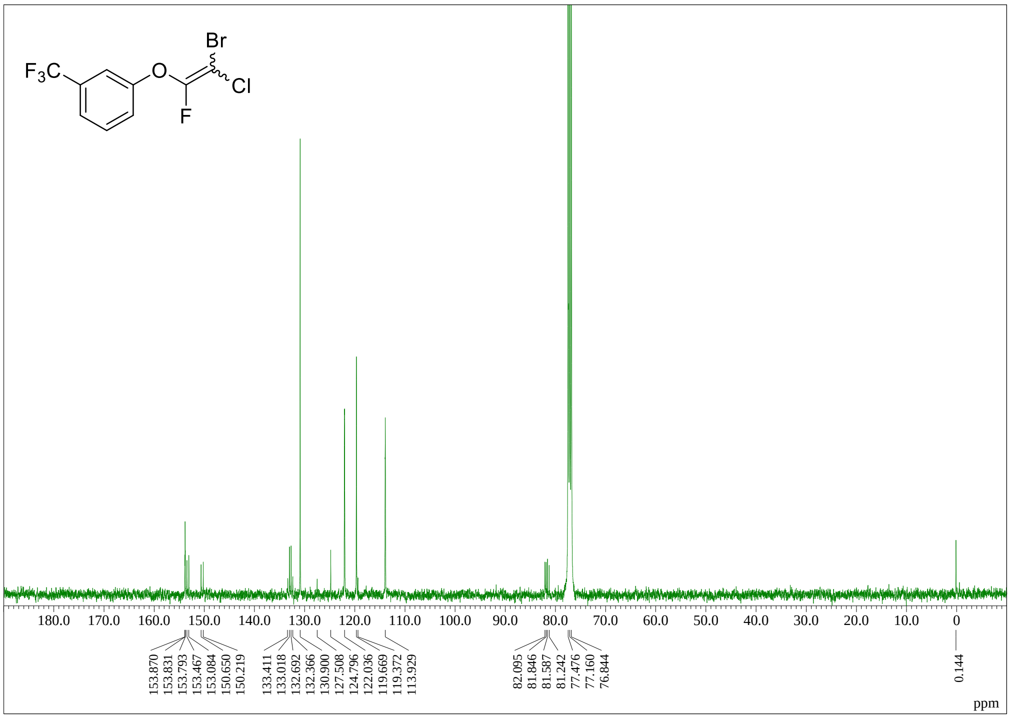


$^{19}$F NMR of **2h**

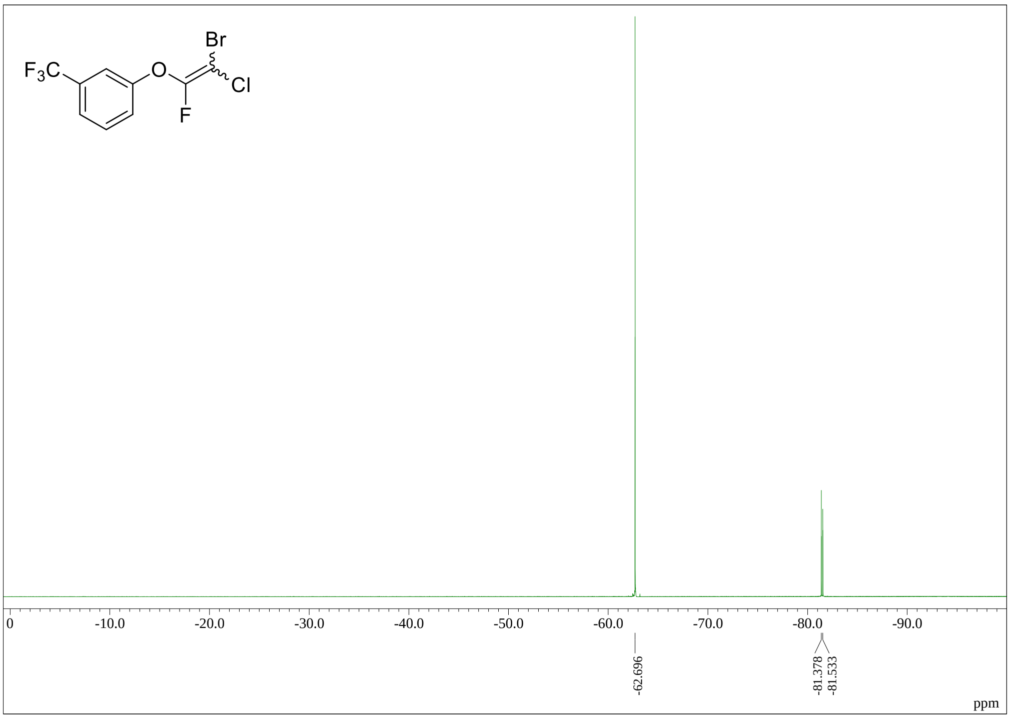

**2-Bromo-2-chloro-1-fluoroethenyl *p*-nitrophenyl ether (2i)**

$^{1}$H NMR of **2i**

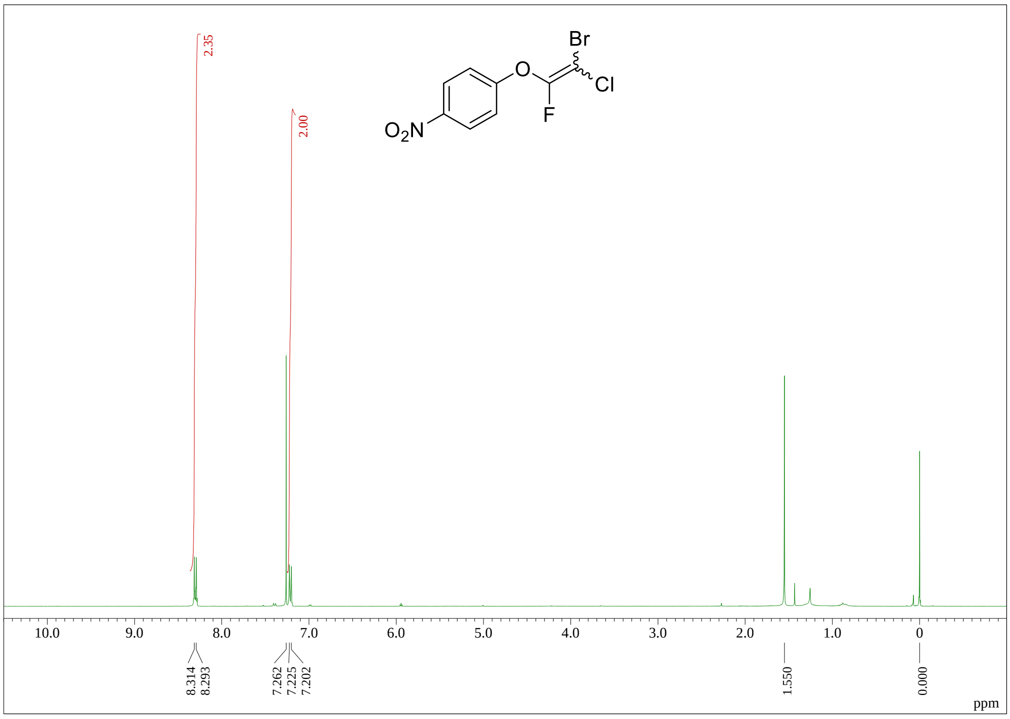


$^{13}$C NMR of **2i**

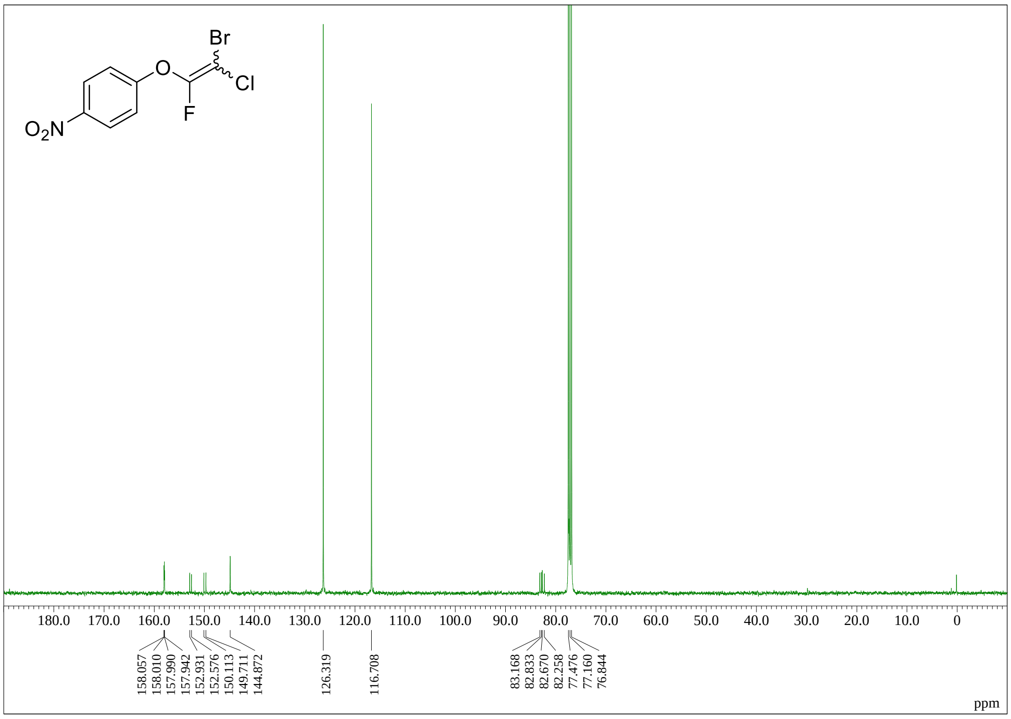

$^{19}$F NMR of **2i**

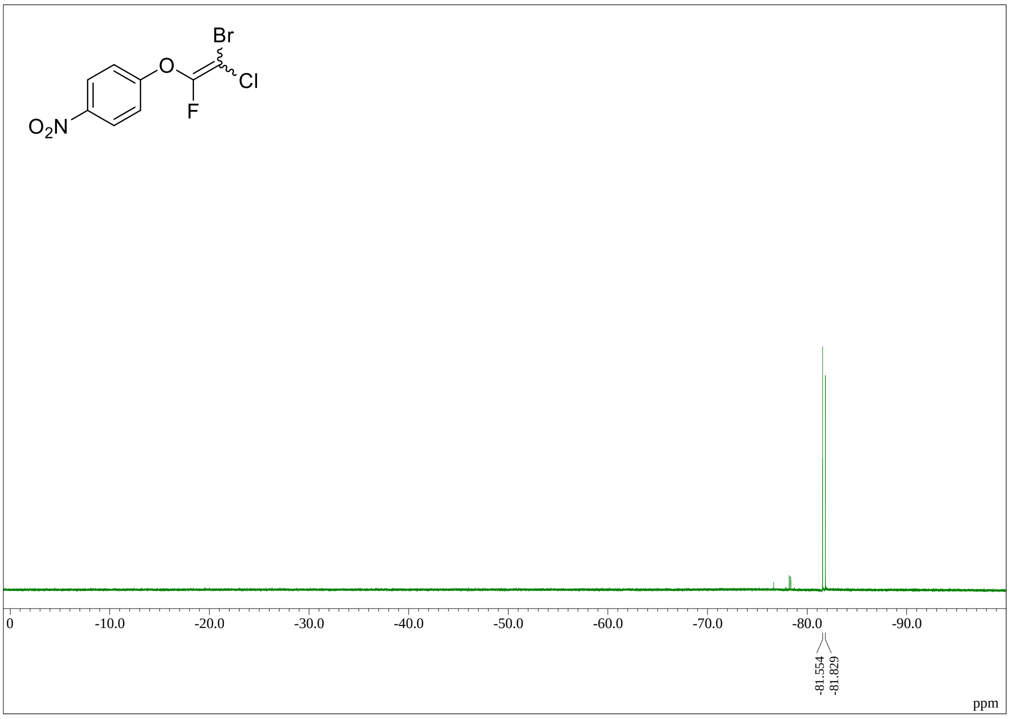


## ***o*-Allylphenyl 2-bromo-2-chloro-1-fluoroethenyl ether (2j)**

$^{1}$H NMR of **2j**

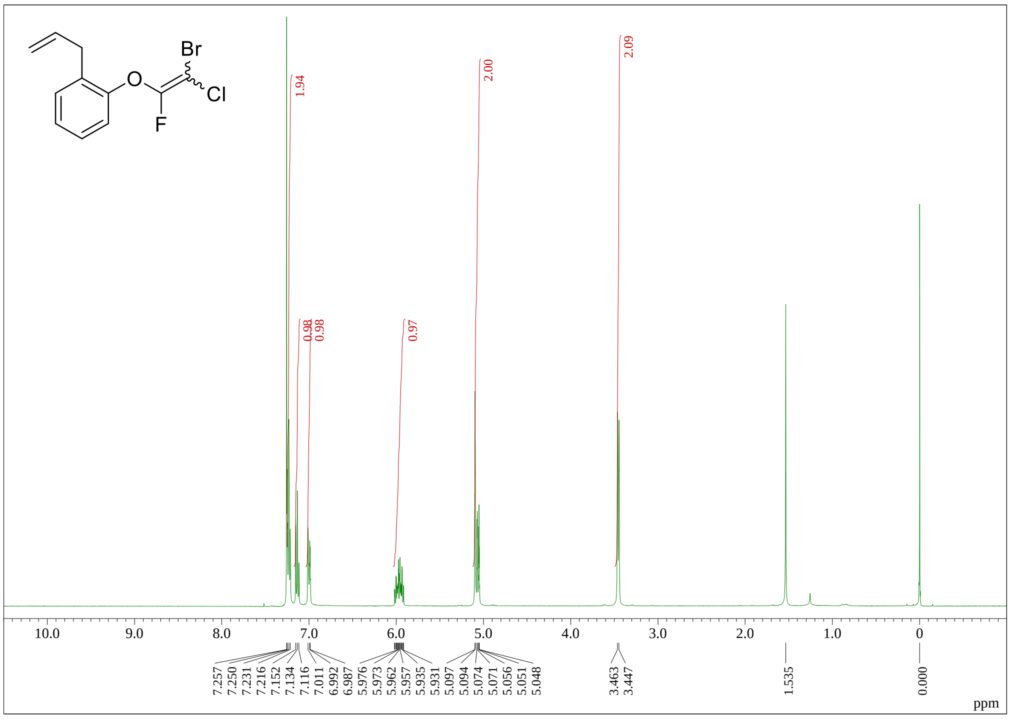

$^{13}$C NMR of **2j**

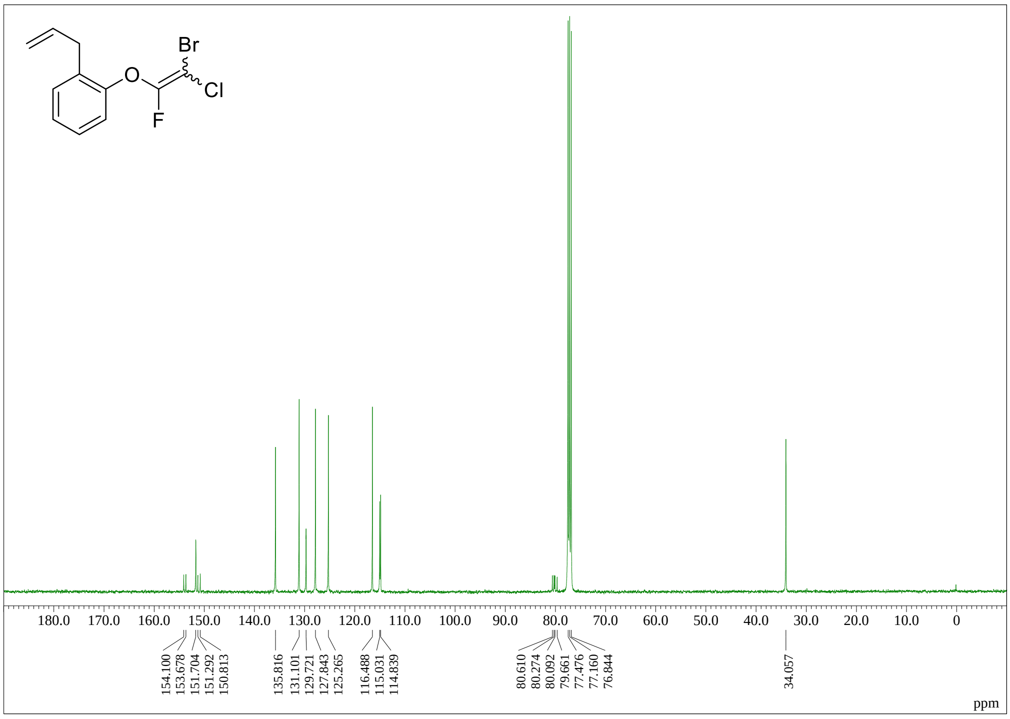


$^{19}$F NMR of **2j**

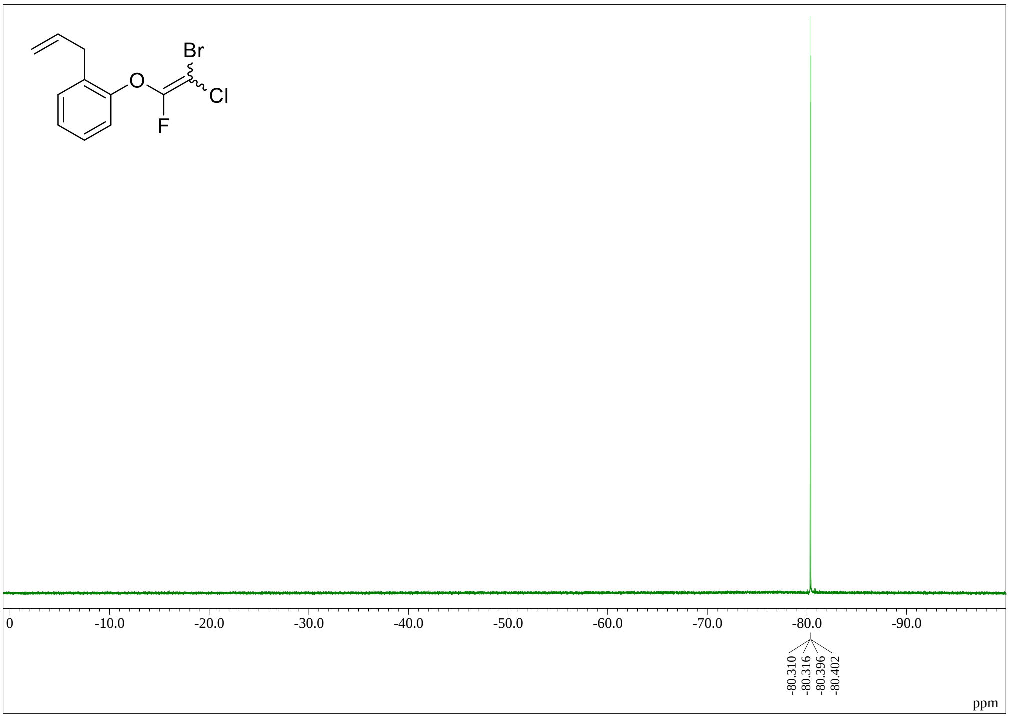

## 2-Bromo-2-chloro-1,1-difluoroethyl *o*-propenylphenyl ether (2k)

$^{1}$H NMR of **2k**

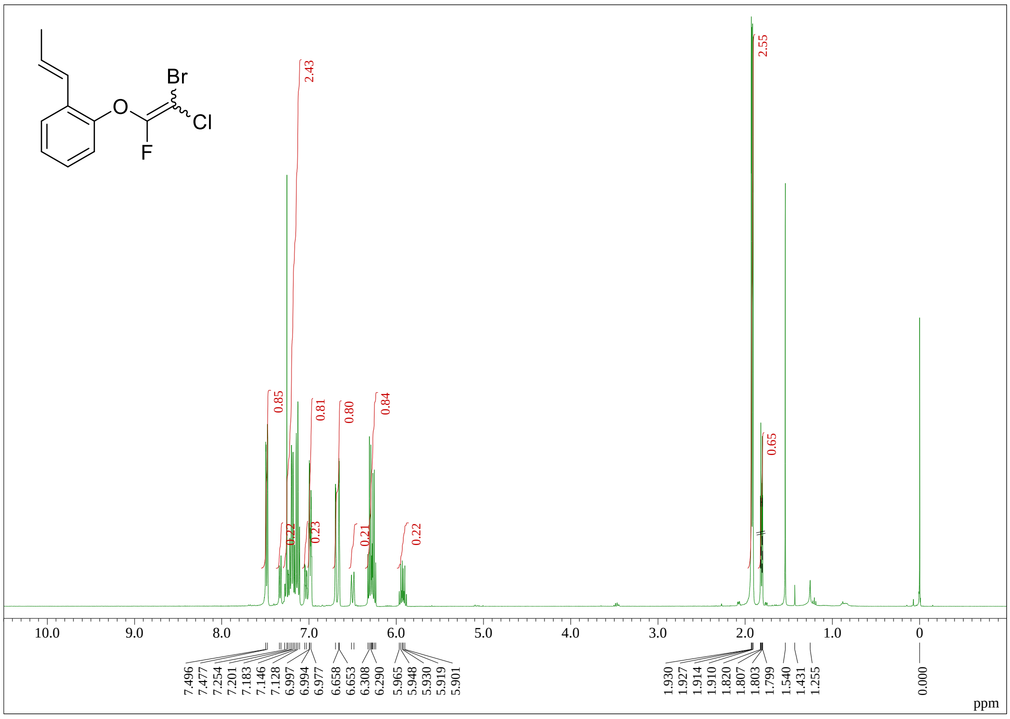

$^{13}$C NMR of **2k**

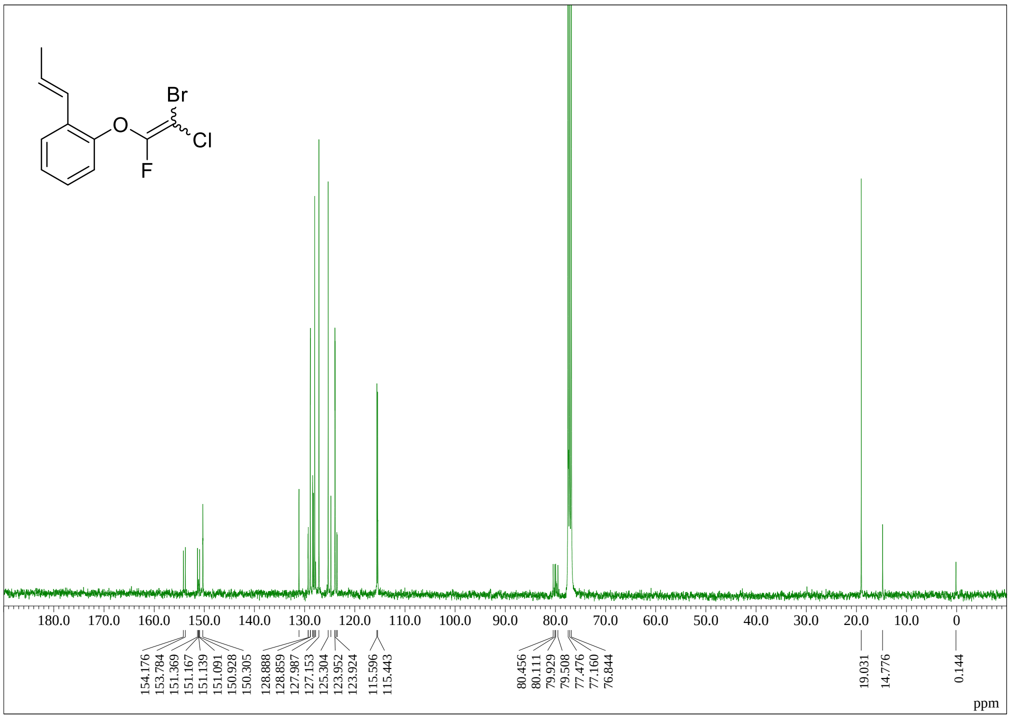

$^{19}$F NMR of **2k**

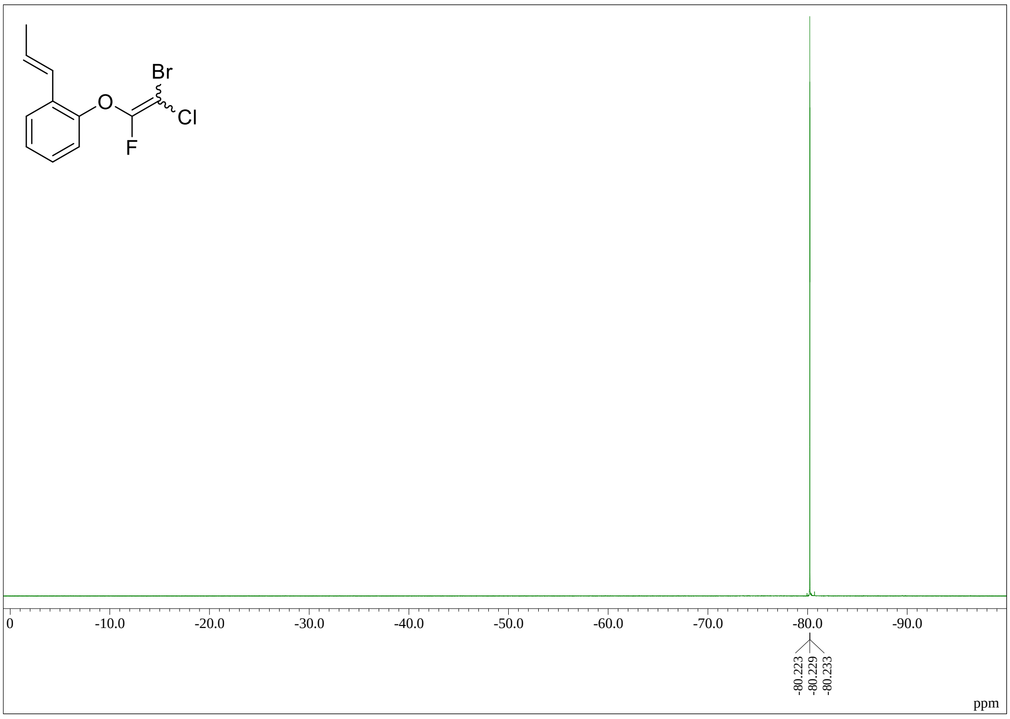


## **(*E*)-2'-(2-Bromo-2-chloro-1-fluoroethenyloxy)-chalcone (2l)**

$^{1}$H NMR of **2l**

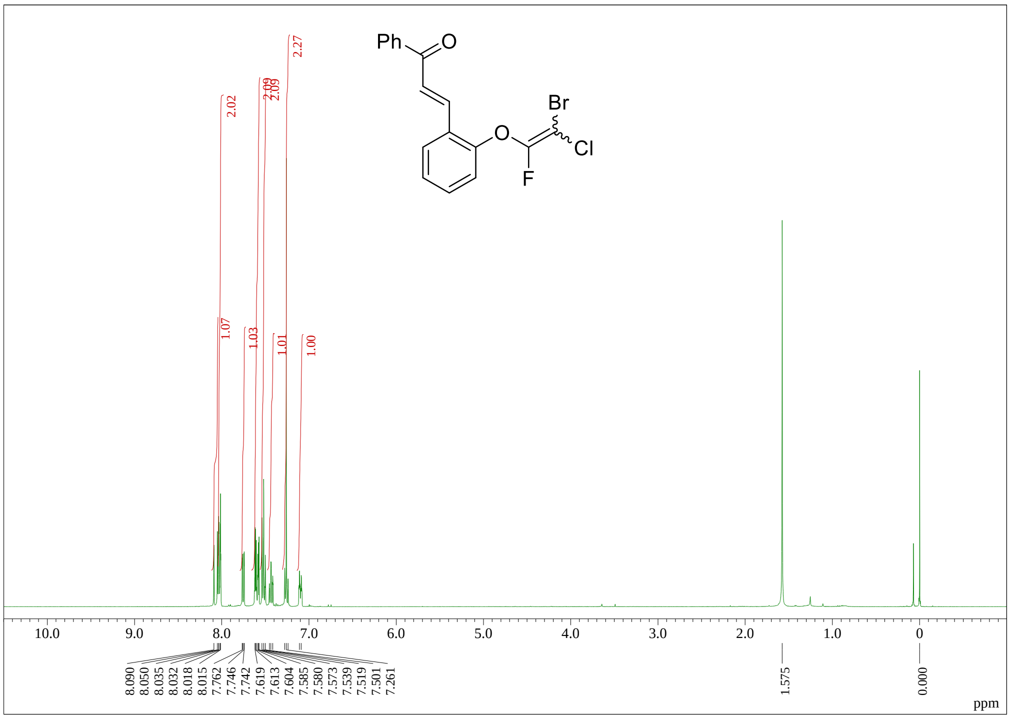

$^{13}C$ NMR of **2l**

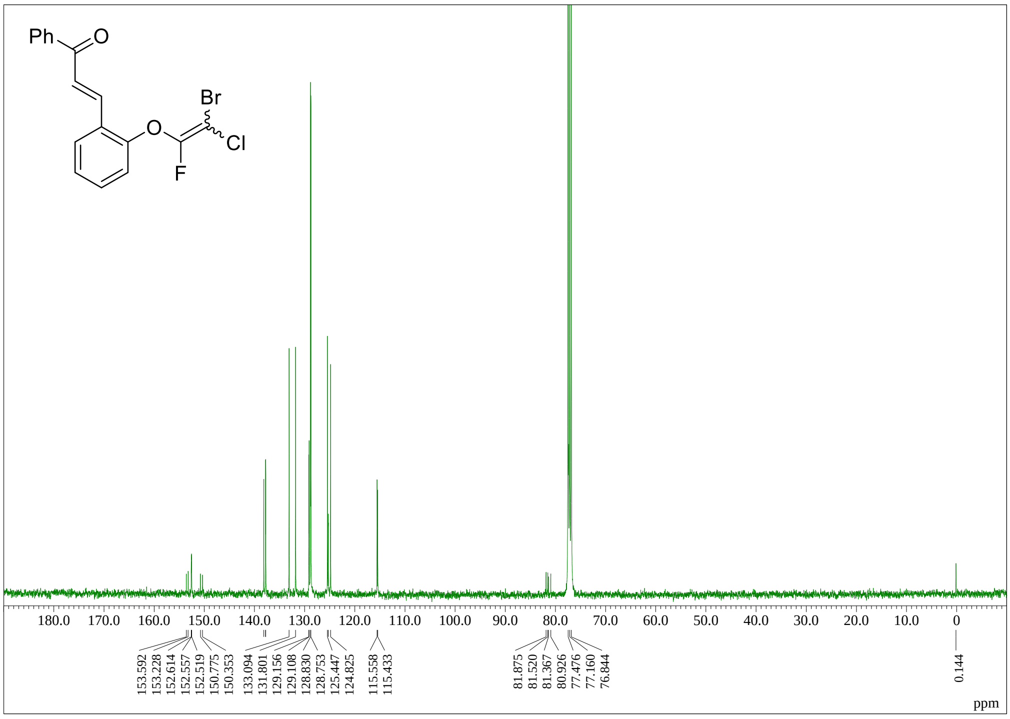


$^{19}F$ NMR of **2l**

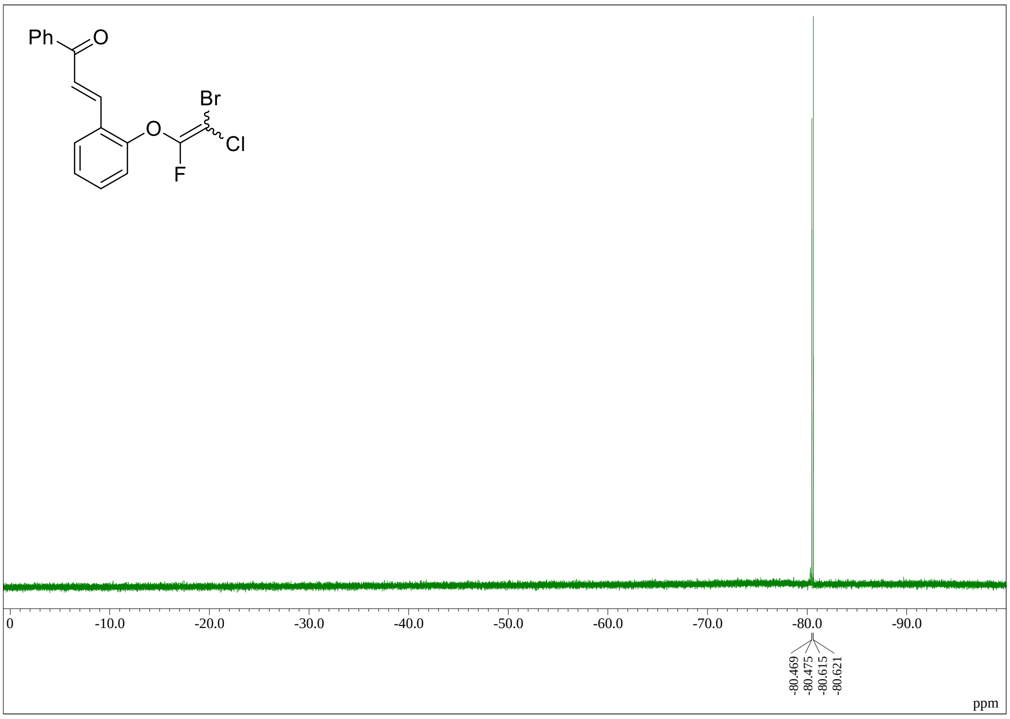

## 2-Bromo-2-chloro-1-fluoroethenyl *p*-formylphenyl ether (2m)

$^{1}$H NMR of **2m**

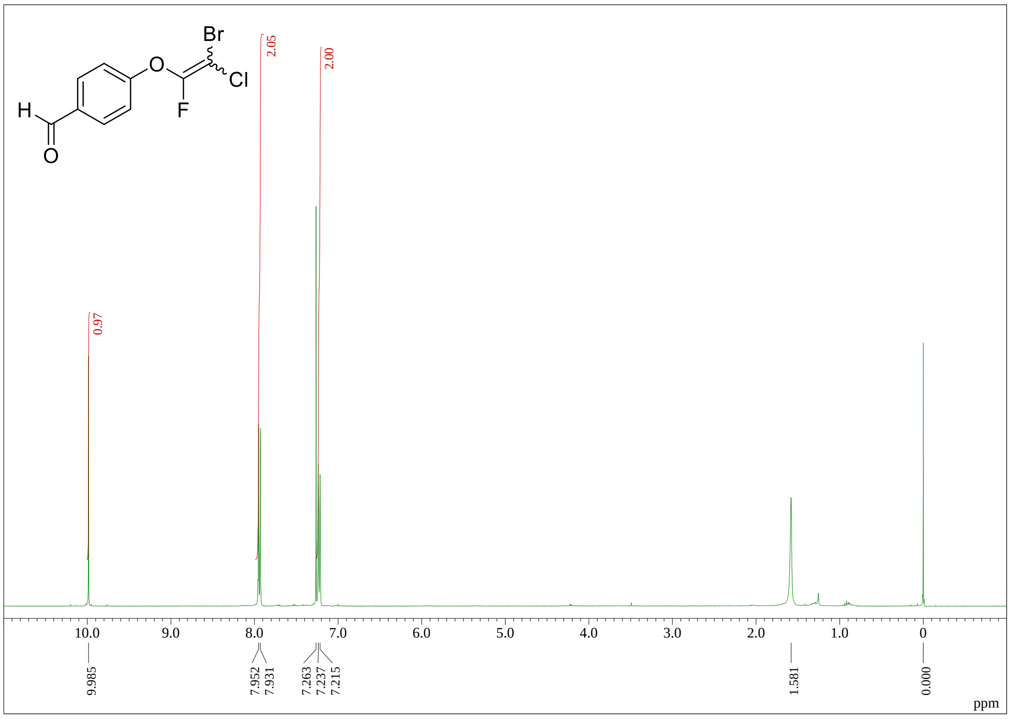


$^{13}$C NMR of **2m**

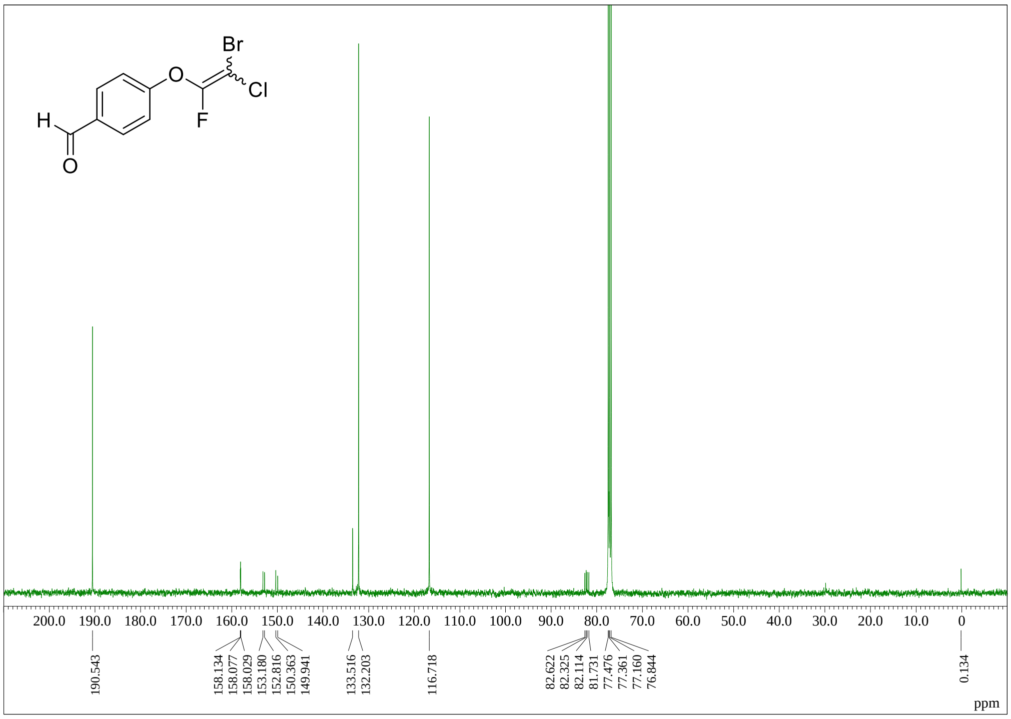

$^{19}$F NMR of **2m**

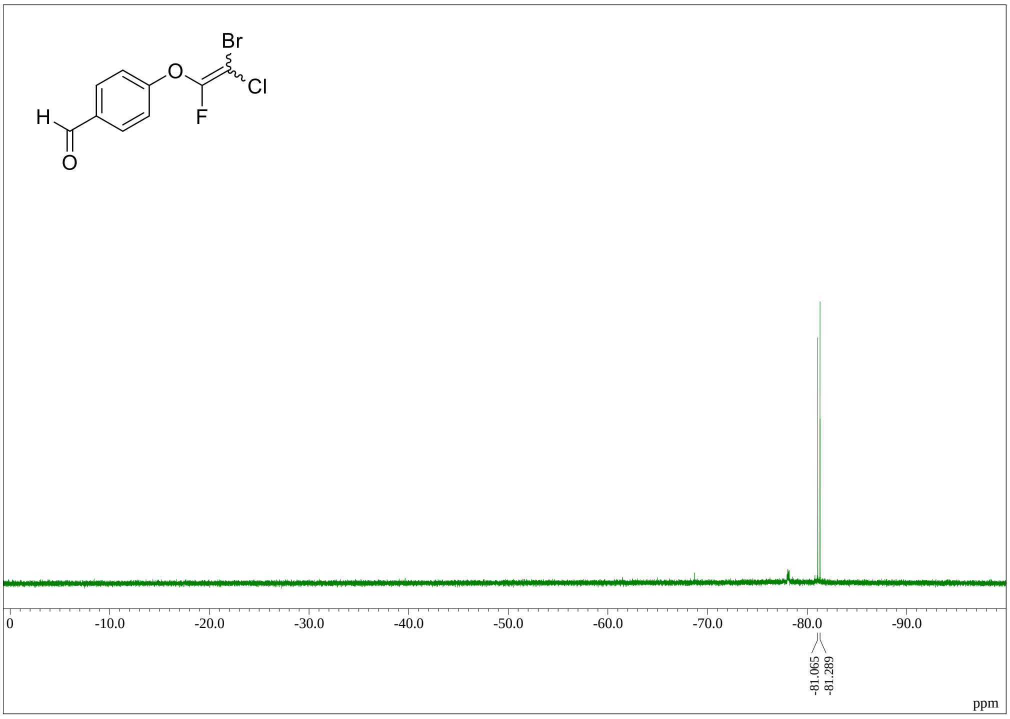


## 2-Bromo-2-chloro-1-fluoroethenyl *p*-ethoxycarbonylphenyl ether (2n)

$^{1}$H NMR of **2n**

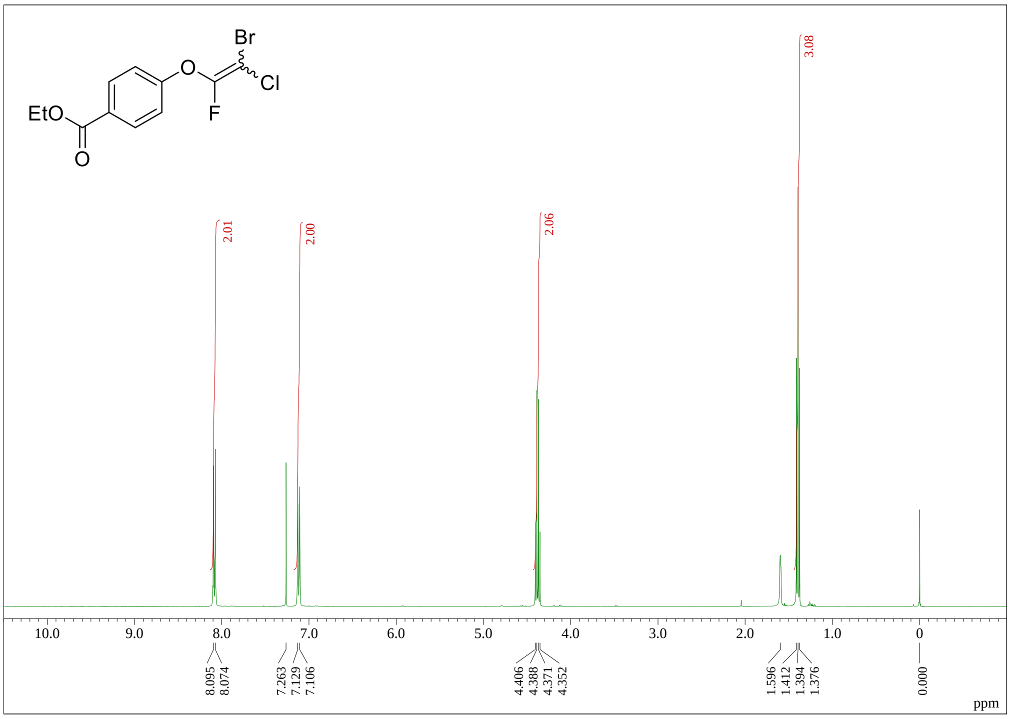

$^{13}$C NMR of **2n**

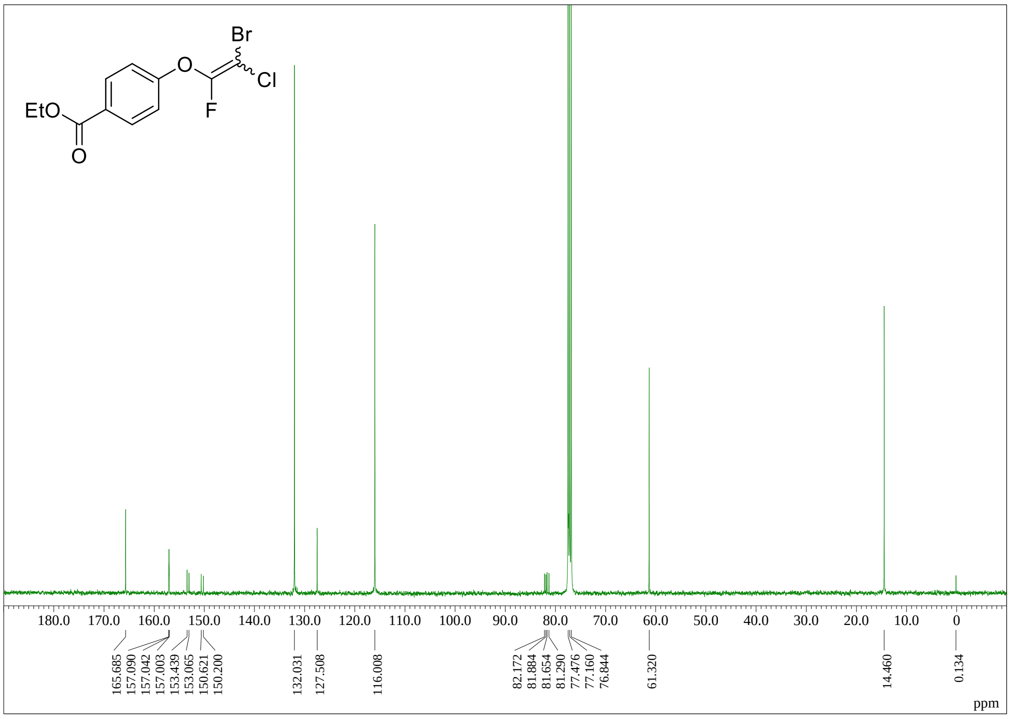


$^{19}$F NMR of **2n**

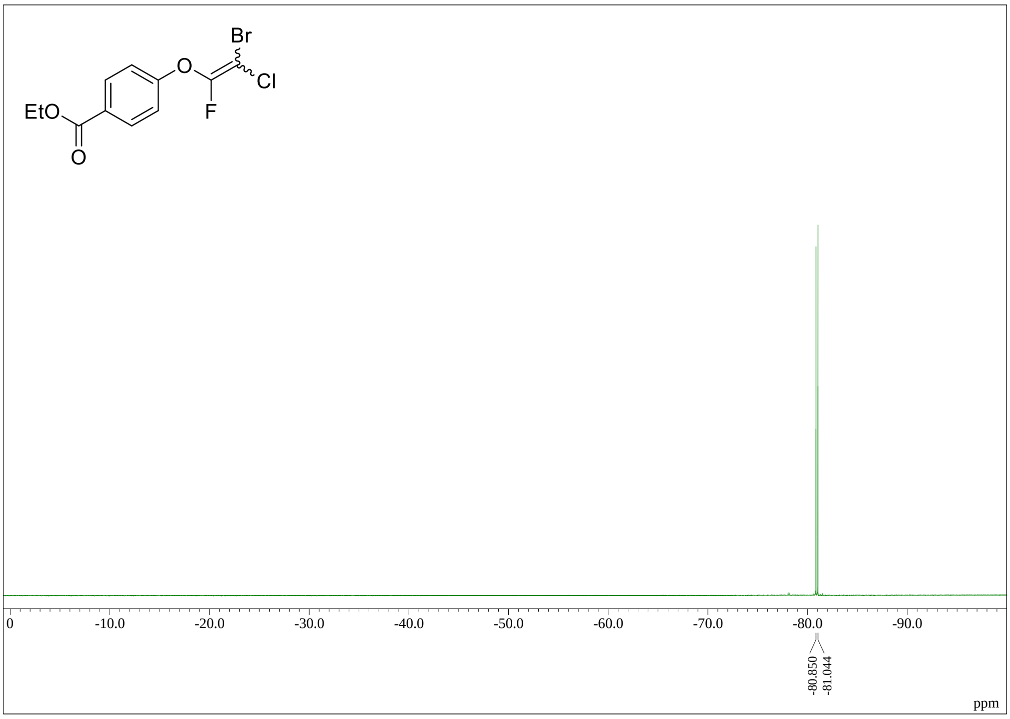

## *m*-Aminophenyl 2-bromo-2-chloro-1-fluoroethenyl ether (2o)

$^{1}$H NMR of **2o**

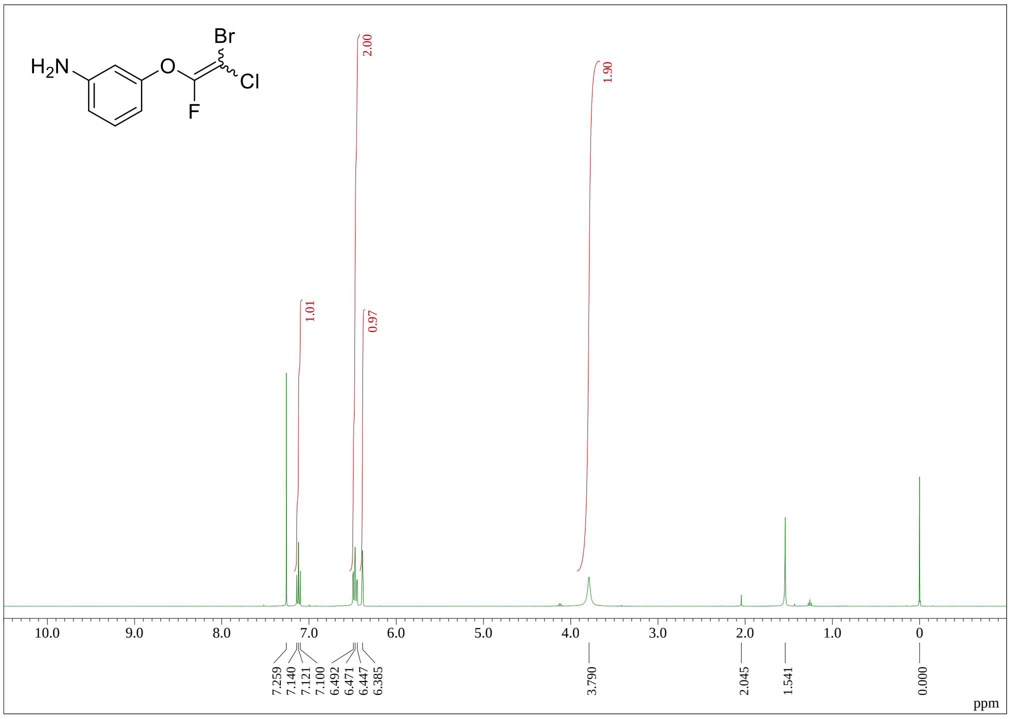


$^{13}$C NMR of **2o**

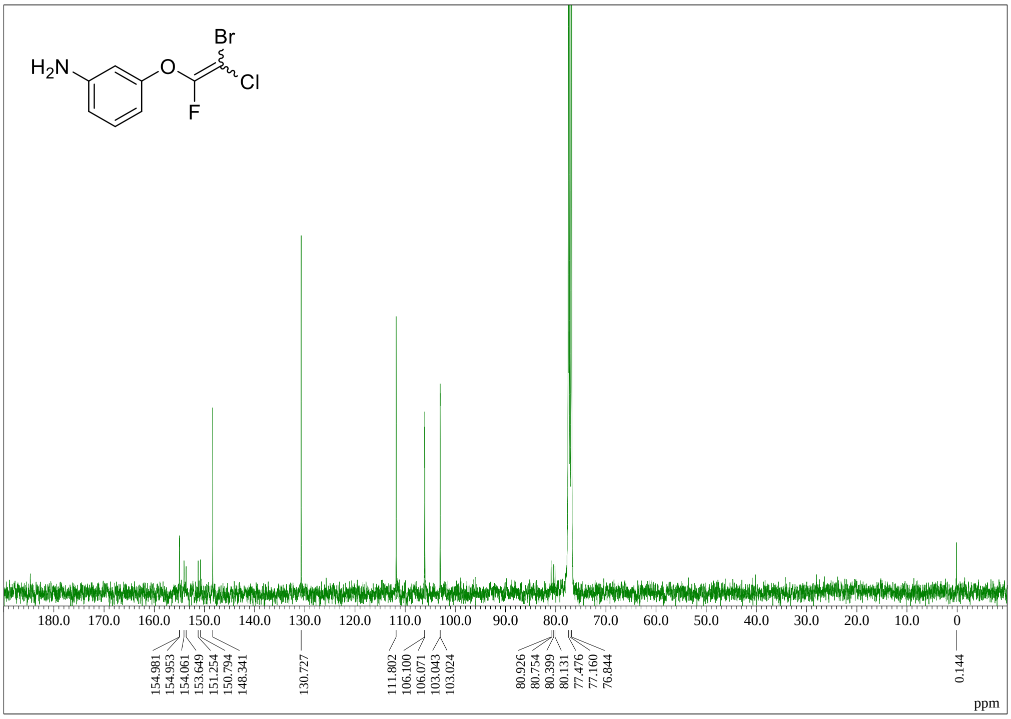

$^{19}$F NMR of **2o**

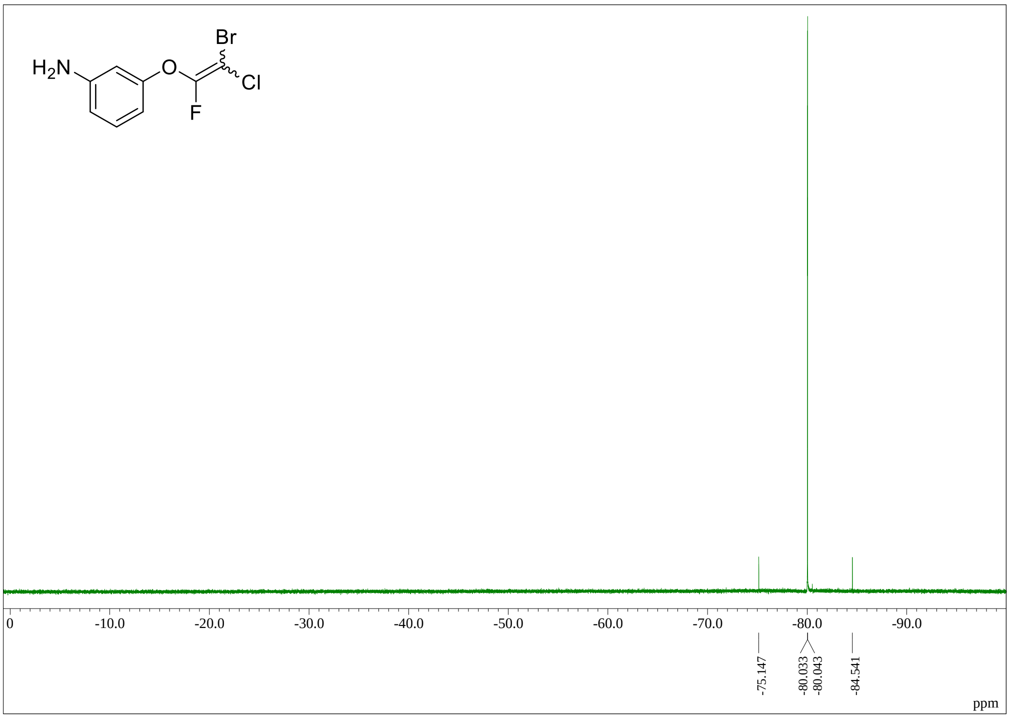


## 2-Bromo-2-chloro-1-fluoroethenyl *o*-iodophenyl ether (2p)

$^{1}$H NMR of **2p**

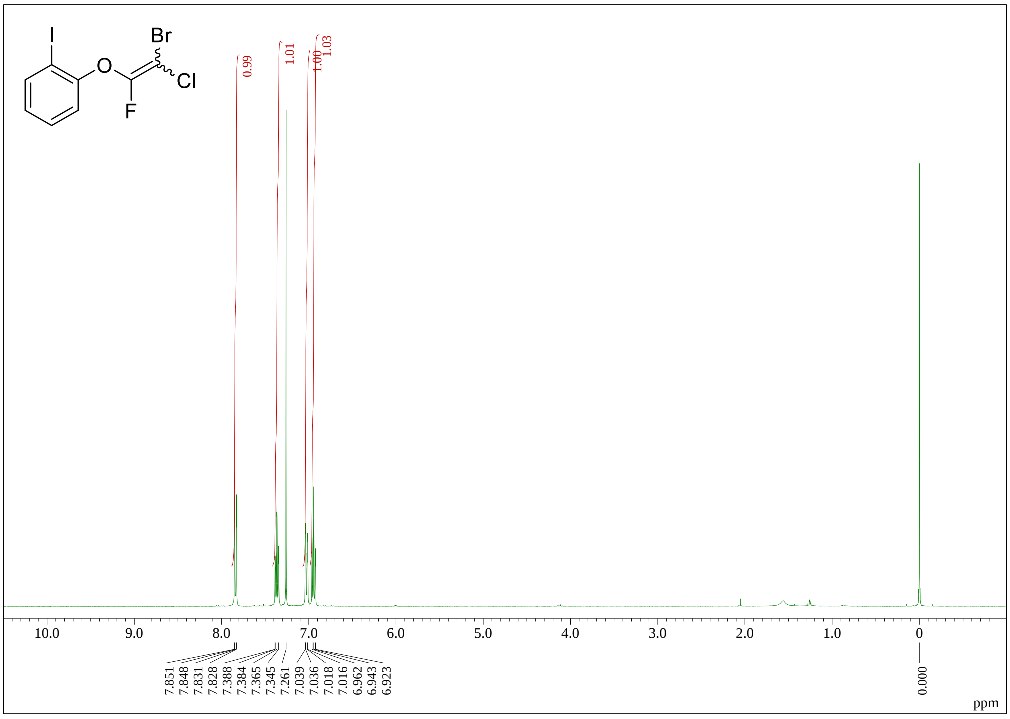

$^{13}C$ NMR of **2p**

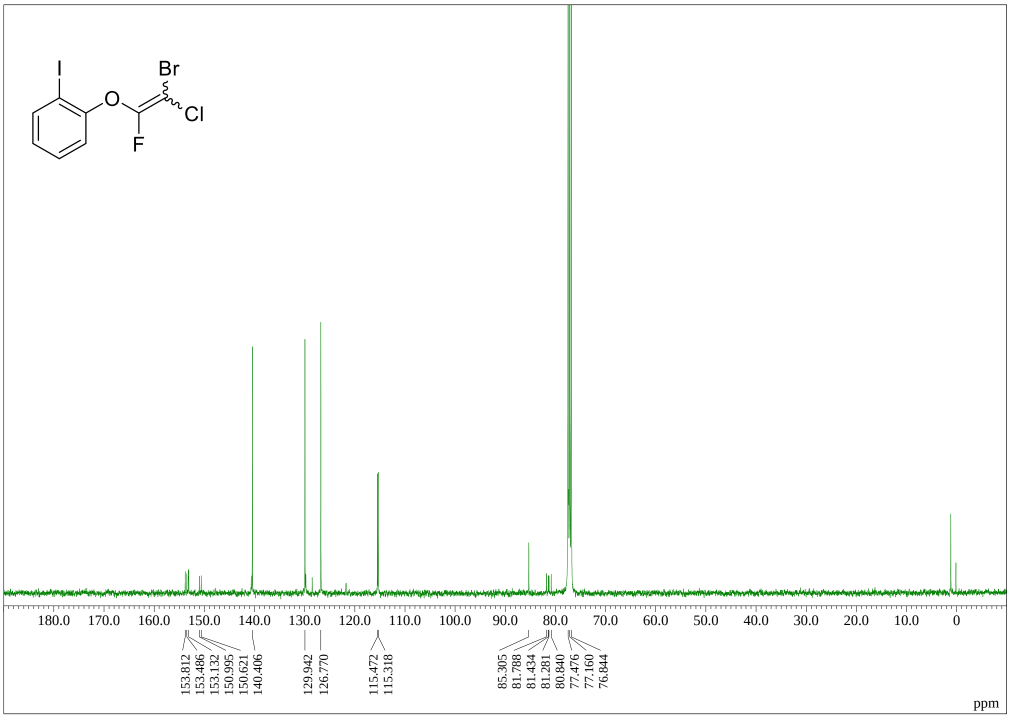


$^{19}F$ NMR of **2p**

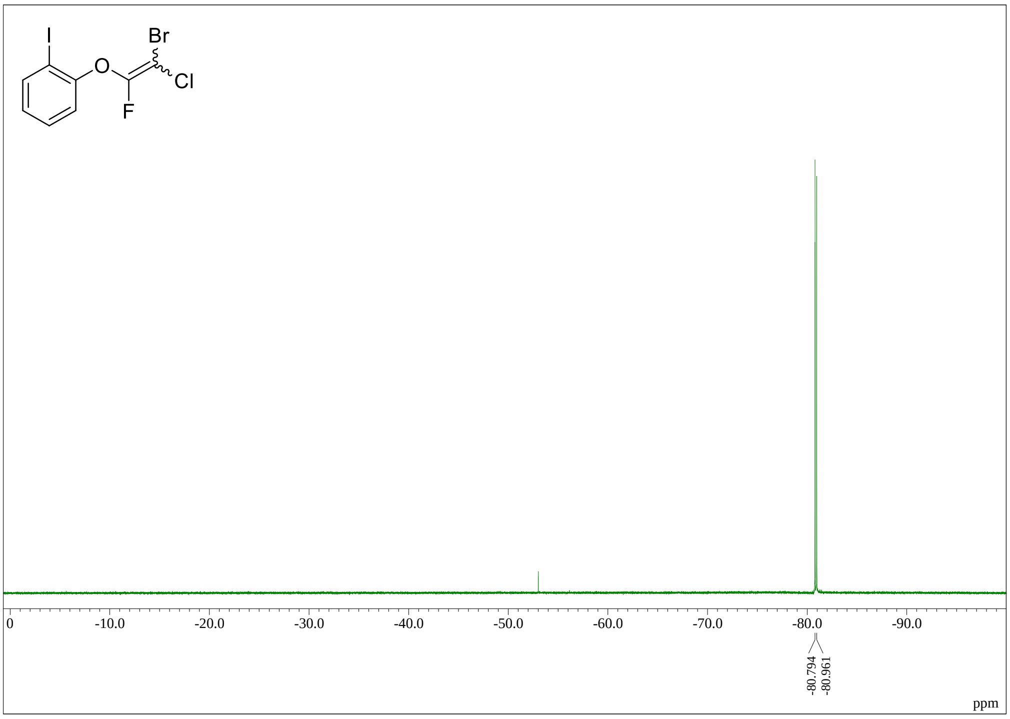

## (3-chloro-4-fluoro-4-phenoxybut-3-en-1-yn-1-yl)trimethylsilane (7)

$^{1}H$ NMR of **7**

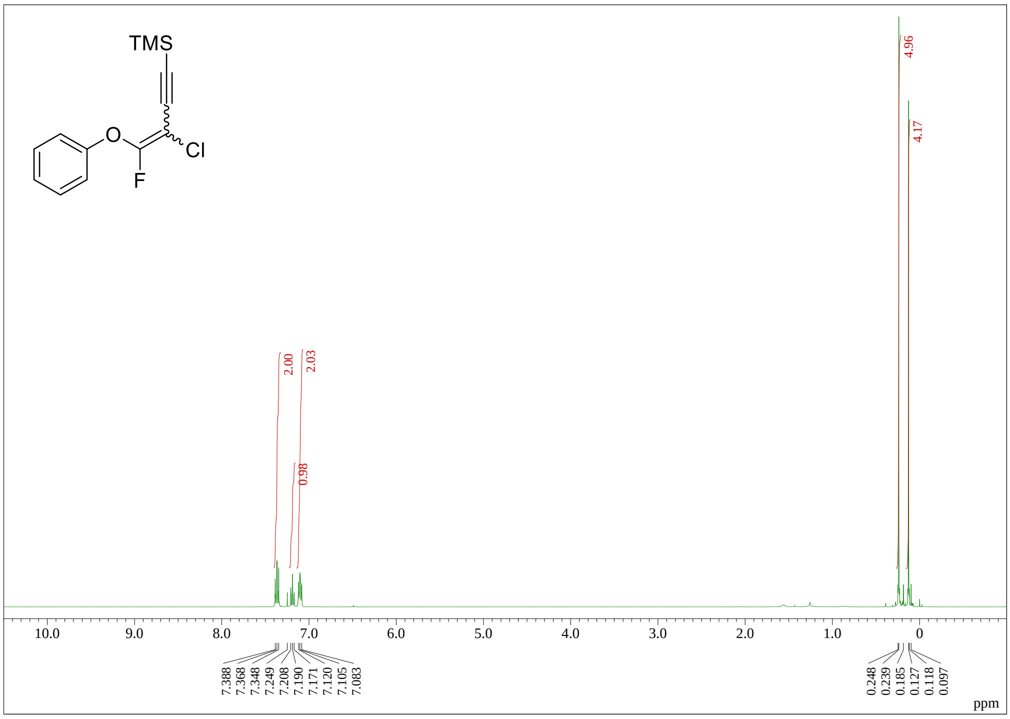


$^{13}C$ NMR of **7**

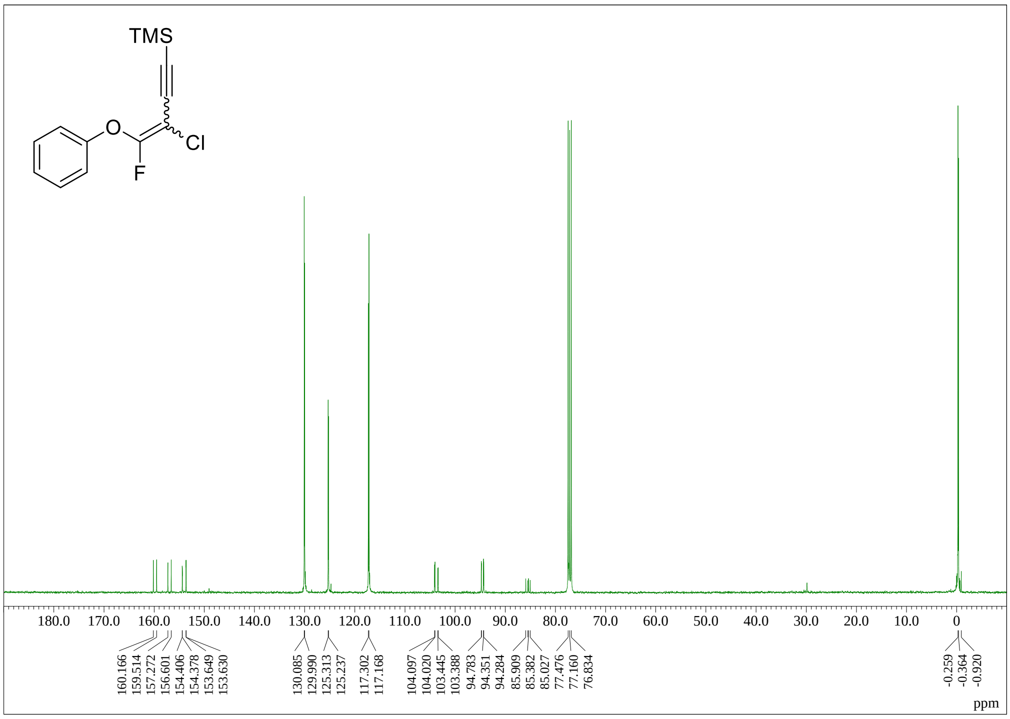

$^{19}$F NMR of **7**

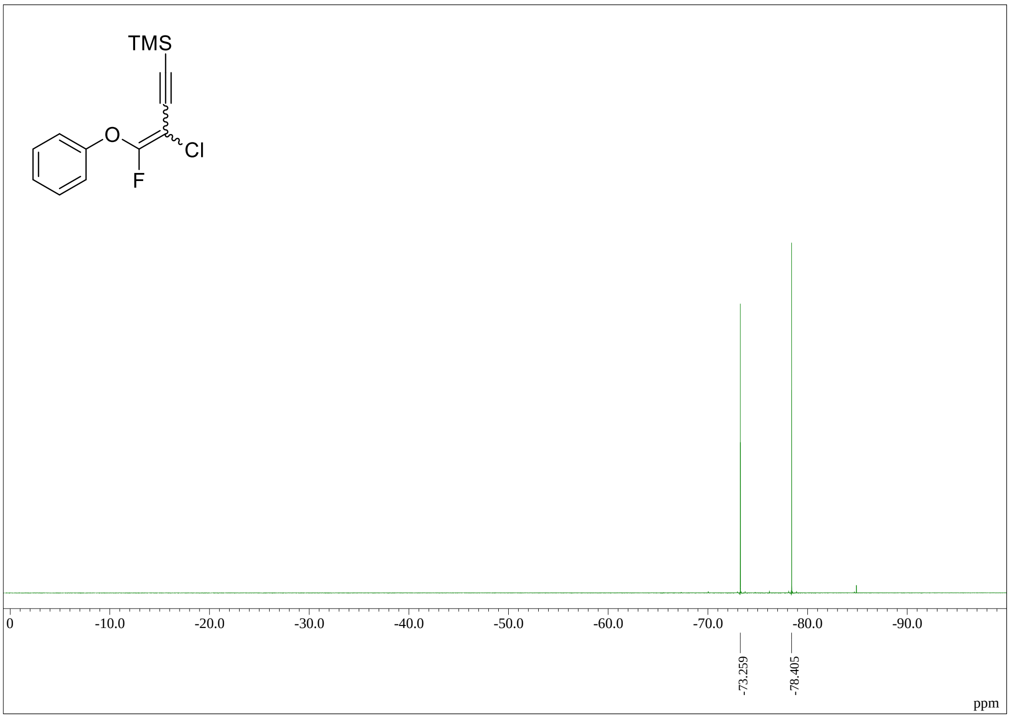

Supplement: File 1 — Characterization data for 2b–p and copies of 1H, 13C, and 19F NMR spectra. [file Beilstein_J_Org_Chem-18-1567-s001.pdf]
